# Aberrant expression and localization of the RAP1 shelterin protein contribute to age-related phenotypes

**DOI:** 10.1371/journal.pgen.1010506

**Published:** 2022-11-28

**Authors:** Amanda J. Stock, Ross A. McDevitt, Chandrakala Puligilla, Yajun Wang, Yongqing Zhang, Kun Wang, Chongkui Sun, Kevin G. Becker, Elin Lehrmann, William H. Wood, Yi Gong, Mohammad Aqdas, Myong-Hee Sung, Victoria Hoffmann, Chengyu Liu, Myriam Gorospe, Lea Harrington, Luigi Ferrucci, Yie Liu

**Affiliations:** 1 Laboratory of Genetics and Genomics, National Institute on Aging/National Institutes of Health, Baltimore, Maryland, United States of America; 2 Comparative Medicine Section, National Institute on Aging/National Institutes of Health, Baltimore, Maryland, United States of America; 3 Laboratory of Molecular Biology and Immunology, National Institute on Aging/National Institutes of Health, Baltimore, Maryland, United States of America; 4 Division of Veterinary Resources, National Institutes of Health, Bethesda, Maryland, United States of America; 5 Transgenic Core Facility, National Heart, Lung, and Blood Institute/National Institutes of Health, Bethesda, Maryland, United States of America; 6 Institute for Research in Immunology & Cancer, Marcelle-Coutu Pavilion, Université de Montréal, Montreal, Quebec, Canada; 7 Translational Gerontology Branch, Biomedical Research Center, National Institute on Aging/National Institutes of Health, Baltimore, Maryland, United States of America; HudsonAlpha Institute for Biotechnology, UNITED STATES

## Abstract

Short telomeres induce a DNA damage response (DDR) that evokes apoptosis and senescence in human cells. An extant question is the contribution of telomere dysfunction-induced DDR to the phenotypes observed in aging and telomere biology disorders. One candidate is RAP1, a telomere-associated protein that also controls transcription at extratelomeric regions. To distinguish these roles, we generated a knockin mouse carrying a mutated *Rap1*, which was incapable of binding telomeres and did not result in eroded telomeres or a DDR. Primary *Rap1* knockin embryonic fibroblasts showed decreased RAP1 expression and re-localization away from telomeres, with an increased cytosolic distribution akin to that observed in human fibroblasts undergoing telomere erosion. *Rap1* knockin mice were viable, but exhibited transcriptomic alterations, proinflammatory cytokine/chemokine signaling, reduced lifespan, and decreased healthspan with increased body weight/fasting blood glucose levels, spontaneous tumor incidence, and behavioral deficits. Taken together, our data present mechanisms distinct from telomere-induced DDR that underlie age-related phenotypes.

## Author summary

As we age, special structures at the ends of our chromosomes, known as telomeres, gradually get shorter. Critically short telomeres activate pathways that disrupt cell division and function or induce cell death. We sought to address the extent to which, as telomeres shorten, telomeric proteins might relocate to other regions of the cell and thus alter cellular function through a telomere-independent mechanism. We chose to focus on the telomere-associated protein, RAP1, since RAP1 is primarily a telomeric protein, but is also found in other regions of the cell where it affects gene expression. To determine the effects of RAP1 distal to telomeres, we generated a *Rap1* mutant mouse model in which RAP1 does not localize at telomeres. Cells derived from this model showed decreased RAP1 expression and increased distribution of RAP1 in the cytosol, similar to that observed in human fibroblasts undergoing telomere attrition. *Rap1* mutant mice exhibited changes in gene expression, inflammation, glucose metabolism, behavior, and decreased lifespan and healthspan. These findings support two important functional roles for RAP1, telomeric and extratelomeric, and suggests that both these respective functions may be integral to aging phenotypes.

## Introduction

Telomeres are chromosome termini-capping structures consisting of tandem DNA nucleotide repeats and a six-protein shelterin complex that includes the repressor/activator protein 1 (RAP1) [1]. Loss of telomere repeats or loss of protection by the shelterin complex can evoke an ataxia-telangiectasia mutated (ATM)- or ataxia-telangiectasia and Rad3-related (ATR)-kinase dependent DNA damage response (DDR), which results in telomere dysfunction-induced foci (TIF) formation and drives cell death or senescence [1]. In humans, telomere shortening is a hallmark of aging [2]. Emerging evidence suggests a crucial role for critically short telomere-induced DDR in cellular dysfunction in telomere biology disorders [3]. However, a causal mechanism elicited by telomere erosion in age-related pathologies, such as cancer, diabetes mellitus, and neurodegeneration remains elusive.

RAP1 is the most conserved telomeric protein from yeast to humans [4]. Mammalian RAP1 relies on its interaction with the telomere repeat binding factor 2 (TRF2) to localize to telomeres [4]. The hydrophobic residues of isoleucine 318 and phenylalanine 336 in RAP1 have been shown to mediate RAP1 and TRF2 interactions in humans [5]. In mice, mutation of isoleucine 312 to an arginine in RAP1 disrupts its interaction with TRF2 and thereby its telomeric localization [6]. Murine RAP1 appears dispensable for telomere end protection. Unlike other shelterin null mice, *Rap1* null mice are viable and fertile [7,8] and lack a telomere dysfunction-induced DDR or chromosome end-to-end fusions [7,9,10]. Conversely, loss of *Rap1* exacerbates telomere shortening and dysfunction in the context of telomerase deficiency [10] or senescence [11]. *Rap1* deficiency also impacts telomere recombination when telomeres have lost protection by key shelterin proteins or TRF2-mediated telomere wrapping/topology [7,12,13].

Although mammalian RAP1 is primarily telomeric, it exerts extratelomeric functions that include transcriptional repression/activation of genes involved in metabolism and cancer [6,8,14–16]. Localization of RAP1 to extratelomeric genomic loci is telomere length-dependent, as progressive telomere shortening leads to re-localization of RAP1 from telomeres to extratelomeric genomic sites [10]. RAP1 influences gene transcription independently of TRF2-mediated binding to telomeres [6,14,17]. RAP1 appears to impact gene transcription through its interactions with proteins distal to telomeres, including epigenetic modifiers [17] as well as the IkappaB kinase in the cytosol [16], which activates Nuclear Factor-kappa B (NF-kB), a master transcription factor that plays fundamental roles in many signaling cascades [18]. *Rap1* null mice also exhibit telomere-independent pathological changes, including obesity with increased white adipose tissue deposition, liver steatosis, and glucose intolerance [6,8]. These results and other published work in budding yeast [19,20] show that RAP1 is involved in cellular functions in addition to a role in telomere maintenance.

Given the age-associated decline in telomere length and TRF2 expression, as well as the decline in telomeric-bound RAP1 as telomeres erode [10,21–23], we hypothesized that an age-associated increase in extratelomeric “free” RAP1 might alter the transcriptome and exacerbate age-associated phenotypes. We therefore generated a knockin mouse model that expressed only the *Rap1* mutant unable to bind TRF2. These *Rap1* knockin mice showed a reduction in RAP1 protein levels and an increased distribution of extratelomeric/cytosolic RAP1. Similar alterations in RAP1 were observed in primary human fibroblasts undergoing telomere attrition. *Rap1* knockin mice exhibited systemic alterations in gene transcription, pro-inflammatory cytokines and chemokines, as well as various age-related phenotypes (i.e., inflammation, metabolic dysfunction, increased tumor incidence, behavioral deficits). Thus, our studies reveal that loss of telomere-bound RAP1 elicits age-associated phenotypes that impact mammalian health and lifespan. These findings suggest that there are important telomeric and non-telomeric roles of RAP1 that may be integral to aging phenotypes and could be modified for therapeutic benefit.

## Results

### Mice expressing extratelomeric RAP1 have decreased lifespans

**Fig 1:**
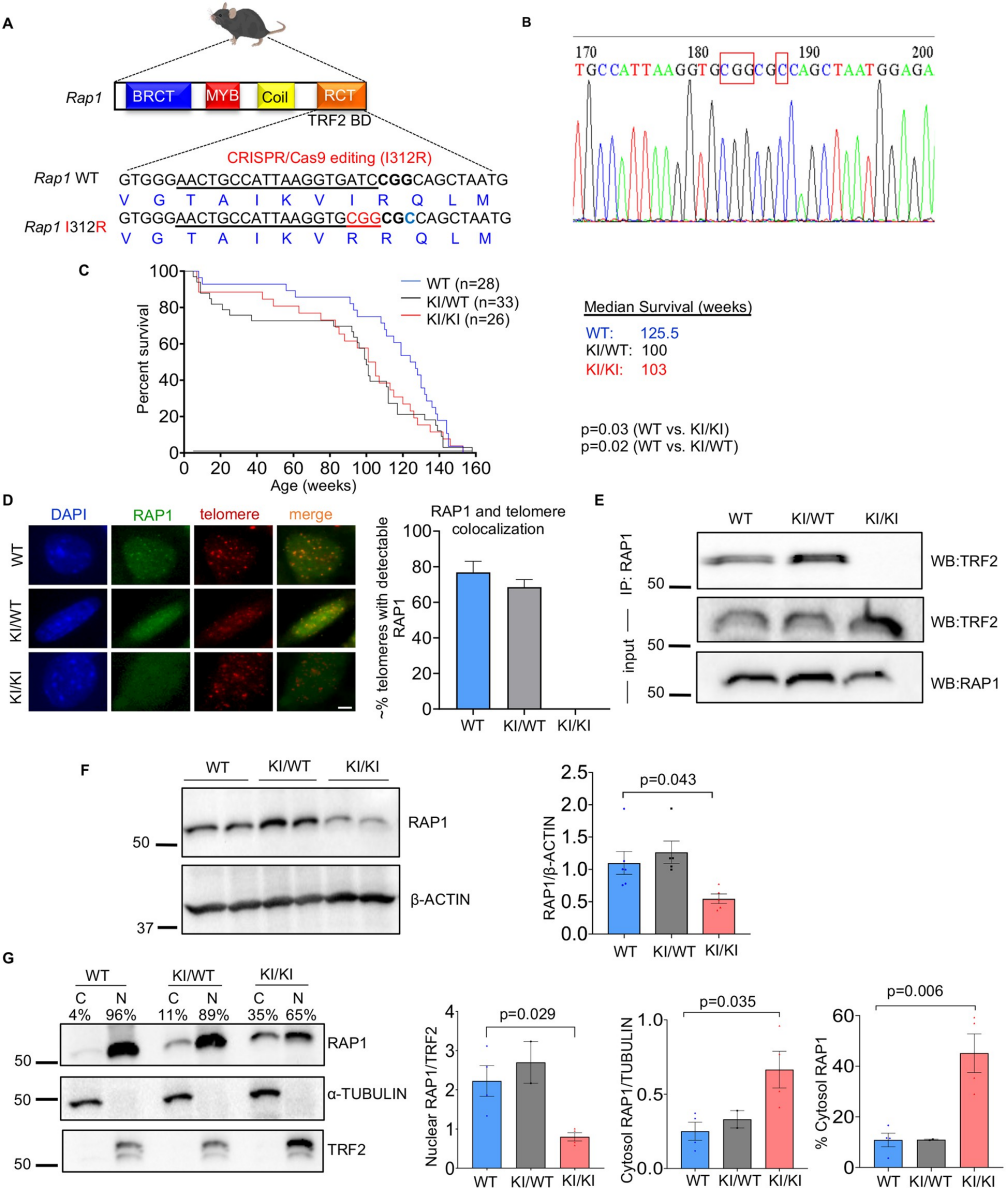
Generation and life span of *Rap1* knockin mice. **(A)** Schematic representation of the generation of a point mutation in the mouse *Terf2ip* (*Rap1*) gene using the CRISPR/Cas9 system. The sgRNA binding sequence is underlined, and the point mutation (red, I312R) was generated at the residue required for RAP1-TRF2 binding within the RCT domain of the WT and *Rap1* knockin allele. *BD*: binding domain. **(B)** Sequence of the *Rap1* mutation. Point mutations are boxed in red, and a single amino acid substitution is shown in the left box. **(C)** Lifespan of WT, *Rap1*^KI/WT^, and *Rap1*^KI/KI^ mice represented by Kaplan-Meier survival curves. Curves for total mice (n = 28 WT, n = 33 *Rap1*^KI/WT^, and n = 26 *Rap1*^KI/KI^, left panel) are displayed. P values were determined using Gehan-Breslow-Wilcoxon tests. **(D)** Representative images and bar graph quantification of nuclei (blue) from primary WT, *Rap1*^KI/WT^, and *Rap1*^KI/KI^ MEFs stained using IF with anti-RAP1 (green) and telomere-FISH (red). Colocalization of RAP1 and telomeres was detected in WT and *Rap1*^KI/WT^, but not in *Rap1*^KI/KI^ MEFs. ~100 nuclei per genotype were analyzed, Scale bar: 5μm. **(E)** Western blot analysis of TRF2 and RAP1 on RAP1 immunoprecipitants and input using whole lysates. **(F)** Western blot and quantification of RAP1 levels in whole-cell lysates. P value was determined by a One-way ANOVA with Tukey’s post-hoc comparisons. n = 6 WT, n = 5 KI/WT, and n = 5 KI/KI primary MEF lines. **(G)** Western blot of cytosol (C) and nuclear (N) fractions probed with anti-RAP1, anti-α-TUBULIN (cytosolic protein), and anti-TRF2 (nuclear protein). Quantifications of RAP1 levels in the nucleus (left), cytosol (middle), and the percentage of RAP1 cytosolic distribution (right) are shown in bar graphs. n = 4 WT, n = 2 *Rap1*^KI/WT^, and n = 4 *Rap1*^KI/KI^ primary MEF lines. P values between WT and *Rap1*^KI/KI^ were determined by student’s unpaired t-tests. All data are mean ± SEM.

To determine the extratelomeric role of RAP1 and its potential contribution to accelerated aging and disease *in vivo*, we used CRISPR/Cas9 editing to generate a *Rap1* knockin (KI) mouse with a point mutation (I312R) in the amino acid previously established to be required for the association of RAP1 with TRF2 and telomeres [5,6] (Fig 1A). The *Rap1* knockin allele was confirmed by Sanger sequencing (Fig 1B). *Rap1* homozygous knockin (*Rap1*^KI/KI^) and heterozygous knockin (*Rap1*^KI/WT^) mice were viable and fertile with a normal Mendelian inheritance pattern, supporting the observation that RAP1 at telomeres is dispensable for murine embryogenesis. Notably, however, *Rap1* knockin mice had decreased lifespans compared to wild-type (WT) littermates (Fig 1C).

### Retention of telomeric and nuclear RAP1 requires its interaction with TRF2

**Fig 2:**
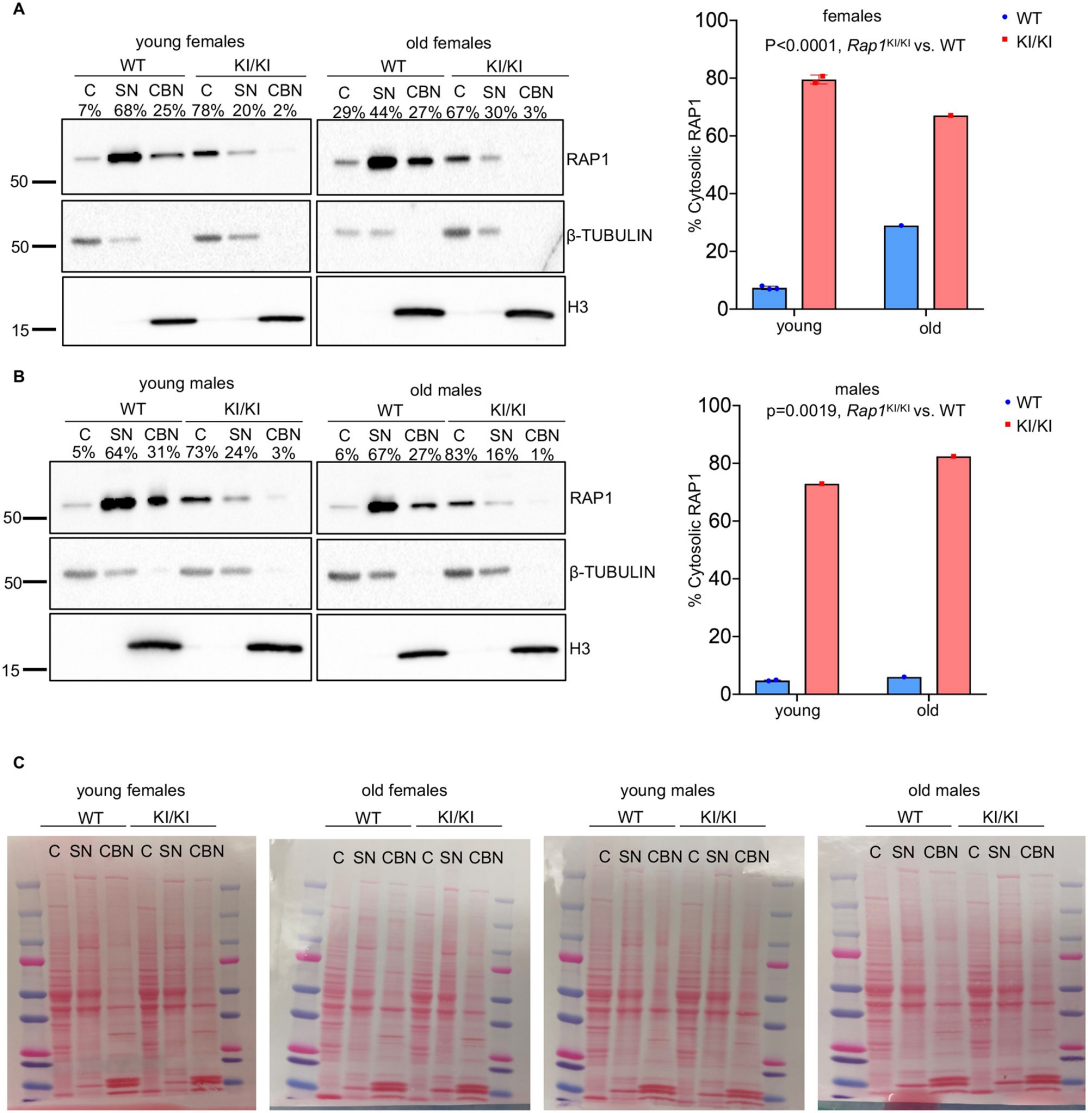
Cytosolic distribution of Rap1 is increased in *Rap1* knockin brain tissues. Western blot of cytosol (C), soluble nuclear (SN), and chromatin bound nuclear (CBN) fractions from whole brains of young and old female **(A)** and male **(B)** WT and *Rap1*^KI/K^ mice probed with anti-RAP1, anti-β-TUBULIN (cytosolic protein), and anti-histone 3 (H3, nuclear protein). Quantifications of RAP1 cytosolic distributions (right) are shown in bar graphs. (C) Ponceau S stains of blots are displayed to clarify size differences of higher molecular weight (RAP1 and β-TUBULIN) compared to lower molecular weight bands (H3) in brain factions. n = 7 WT and n = 5 *Rap1*^KI/KI^ mice. P values were determined by two-way ANOVAs. All data are mean ± SEM.

To assess the anticipated absence of RAP1 at telomeres in *Rap1*^KI/KI^ mice, we performed immunofluorescence-telomere fluorescence in situ hybridization (IF-telomere FISH) on primary mouse embryonic fibroblasts (MEFs). RAP1 co-localized with telomeres in WT and *Rap1*^KI/WT^ MEFs. In *Rap1*^KI/KI^ MEFs, 100% of the cells exhibited diffuse nuclear/cytosolic staining, and no RAP1 was detected at nuclear foci or telomeres (Fig 1D). Anti-RAP1 immunoprecipitation showed that TRF2 was detected in WT and *Rap1*^KI/WT^ MEFs, but not in *Rap1*^KI/KI^ MEFs (Fig 1E). These results confirm that this RAP1 mutant does not bind TRF2 or associate with telomeres [6]. We did not detect changes in *Rap1* mRNA levels in *Rap1*^KI/KI^ MEFs (S1A Fig). Western blotting demonstrated that RAP1 protein levels were decreased in *Rap1*^KI/KI^ MEFs, compared to WT MEFs (Fig 1F), consistent with a previous observation that RAP1 protein levels are reduced in the absence of the RAP1-TRF2 interaction [7]. RAP1 protein levels in *Rap1*^KI/WT^ MEFs were comparable to WT MEFs (Fig 1F). Cell fractionation and western blotting showed increased cytosolic RAP1 in *Rap1*^KI/WT^ and *Rap1*^KI/KI^ MEFs, compared to WT MEFs (Fig 1G). Similar changes in RAP1 were observed in *Rap1*^KI/KI^ mice, *i*.*e*., *Rap1*^KI/KI^ brain tissues showed reduced RAP1 expression and increased cytosolic RAP1 distribution (Fig 2), which was apparent in young and old female and male mice. A modest reduction in RAP1 expression and an increase in cytosolic RAP1, with no marked changes in TRF2 cytosolic distribution, occurred in human primary fibroblasts undergoing telomere attrition during prolonged culture (S2 Fig). We did not detect changes in *Trf2* mRNA (S1B Fig) or TRF2 protein levels (S1C Fig) in MEFs expressing RAP1 I312R or evidence of TRF2 re-localization to the cytosol (Fig 1G), indicating that RAP1 can localize to the cytosol independently of TRF2. In summary, the changes in *Rap1* expression and RAP1 cytosolic distribution that occur in the *Rap1* knockin model are reminiscent of those changes observed during replicative senescence in human cells.

### Loss of telomeric RAP1 does not disrupt telomere maintenance

Mounting evidence suggests that replicative senescence and telomeropathies are triggered by a loss of telomere DNA repeats and a telomere dysfunction-induced DDR [3,24]. Since there is loss of telomeric RAP1 in *Rap1*^KI/KI^ mice, we assessed telomere length and telomere-dysfunction induced DNA damage foci (TIF) formation in primary WT and *Rap1*^KI/KI^ MEFs. Telomere signal intensities in *Rap1*^KI/KI^ MEFs were comparable to WT MEFs by quantitative telomere in situ hybridization (Q-FISH) on metaphase spreads (S3A Fig). Furthermore, we did not observe telomere signal free ends, telomere fragility, chromosome end-to-end fusions, or other gross chromosome abnormalities in *Rap1*^KI/KI^ MEFs (S3A Fig). TIFs were determined by co-localization of γH2AX foci and telomere DNA using IF-telomere FISH [25]. Approximately 1% WT and *Rap1*^KI/KI^ MEFs harbored one TIF per nucleus, and no MEFs showed ≥3 TIFs per nucleus (S3B Fig). Similar results were also observed in WT and *Rap1*^KI/KI^ mouse tissues (S3C Fig). These results are in keeping with prior studies in MEFs, where this *Rap1* mutant did not elicit a telomere-dysfunction-induced DDR [6]. Collectively, *Rap1*^KI/KI^ mice exhibit intact telomere length and capping. Moreover, we measured senescence associated beta galactosidase activity (SA-beta gal) in primary WT and *Rap1*^KI/KI^ MEFs, and no differences in the percentage of SA-beta gal positive cells were observed (S4 Fig). Therefore, the *Rap1*^KI/KI^ mouse model allows us to distinguish phenotypes driven by alterations in RAP1 from those driven by a telomere-induced DDR.

### Systemic gene transcription is disrupted in mice expressing extratelomeric RAP1 *in vivo*

**Fig 3:**
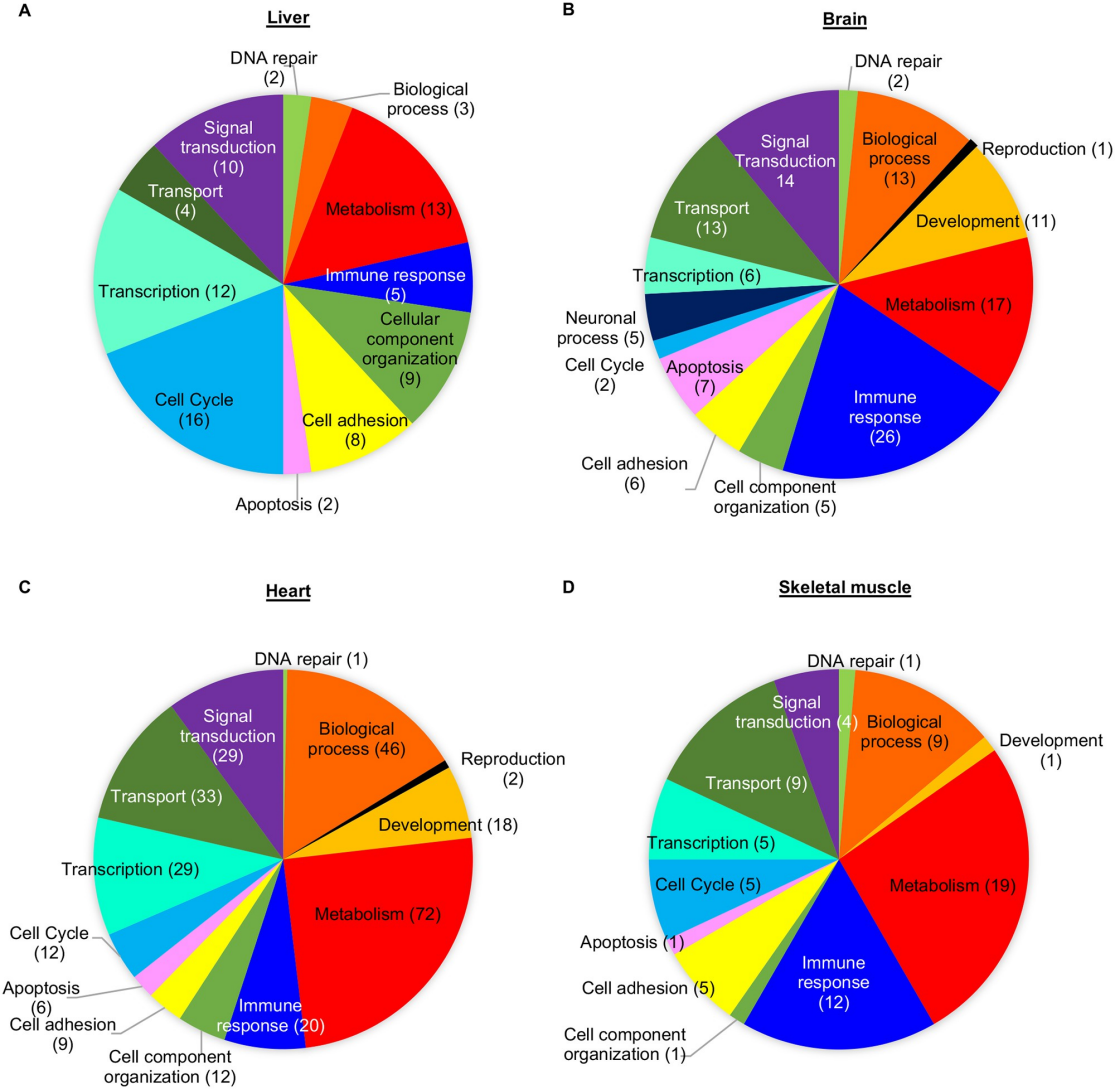
Gene transcription and ontology are altered in *Rap1* knockin male mice. Gene ontology classifications for significantly altered transcripts detected by microarray analysis in the livers **(A)**, brains **(B)**, hearts **(C)**, and skeletal muscles **(D)** of *Rap1*^KI/KI^ males compared to the WT control males.

Previous findings support a role for murine RAP1 in regulating gene transcription, including genes involved in metabolism [6,8,14]. The transcriptional effects of RAP1 can be largely attributed to its extratelomeric role, since the *Rap1* I312R allele almost completely restored the transcriptional profile of *Rap1* null MEFs *in vitro* [6]. We assessed the impact of extratelomeric RAP1 on the transcriptomic profiles in *Rap1*^KI/KI^ mice *in vivo*. Compared to WT littermate controls, a total of 135, 299, 486, and 74 transcripts were significantly altered in the brain, heart, liver, and skeletal muscle of *Rap1*^KI/KI^, respectively (S1 Table). Altered transcripts were observed in the liver, brain, heart, and skeletal muscle in *Rap1*^KI/KI^ young male mice (2–5 months) (S1 Table and Fig 3). Numerous transcripts were altered in the livers of middle-aged *Rap1*^KI/KI^ female mice (12–14 months), although few transcripts were significantly changed in other tissues examined (S1 Table and S5 Fig). Gene ontology/pathway analysis revealed a complete list of significantly deregulated pathways, the majority of which were implicated in metabolism (S2 Table and Fig 3 and S5 Fig). The liver showed the largest number of significantly deregulated mRNAs, corresponding to the downregulation of most metabolic pathways, but these pathways were upregulated in the brain, heart, and skeletal muscle (S2 Table). It is possible that upregulation of mRNAs encoding metabolic factors in these tissues of *Rap1*^KI/KI^ animals acts as a compensatory mechanism to mitigate the downregulation of mRNAs encoding metabolic proteins in the liver. Immune-response pathways were significantly upregulated in all four tissues analyzed, especially in the brain and muscle (S2 Table). Together, our results suggest that the absence of RAP1 at telomeres contributes important functions in the transcriptional regulation of metabolism and the immune-response regulation *in vivo*.

### Mice expressing extratelomeric RAP1 show increased production of pro-inflammatory cytokines and chemokines

RAP1 is detected in the cytoplasm where it activates NF-κB [16], a master transcription factor that is associated with aging and plays fundamental roles in stimulating inflammation and immune responses, and promoting tumorigenesis [18]. *Rap1* deletion results in defective NF-κB activation in mice [16]; however, given the significant increase of cytosolic RAP1 and immune-response gene transcription in the *Rap1*^KI/KI^ mice, we assessed levels of p65 (the major NF-κB subunit) by crossing *Rap1*^KI/KI^ mice with *RelA*-EGFP reporter mice [26]. *In vivo* levels of p65 (RelA) in *RelA*-EGFP mice that expressed WT or *Rap1*^KI/KI^ were determined by flow cytometry on freshly isolated bone marrow cells and splenocytes. A modest increase in the percentage of p65 positive cells (GFP+) and p65 fluorescence intensity was observed in *Rap1*^KI/KI^ compared to WT bone marrow cells and splenocytes (S6A Fig). This result indicates that NF-κB expression is elevated in a small fraction of bone marrow cells and splenocytes *in vivo*. NF-κB activation was also assessed by measuring the levels of p65 in primary WT and *Rap1*^KI/KI^ bone marrow-derived macrophage (BMDM) nuclear extracts by the p65 enzyme-linked immunosorbent assay. The levels of p65 in nuclear extracts from *Rap1*^KI/KI^ BMDMs were slightly increased (S6B Fig). Although activation of p65 was subtle in the absence of any stimuli, various pro-inflammatory cytokines and chemokines, including IL-6 and IL-1β, which are induced by NF-κB activation, were elevated in *Rap1*^KI/KI^ BMDMs relative to WT BMDMs (S3 Table). These results indicate that cytosolic RAP1 could promote NF-κB signaling in *Rap1*^KI/KI^ mice, even under unchallenged conditions.

**Fig 4:**
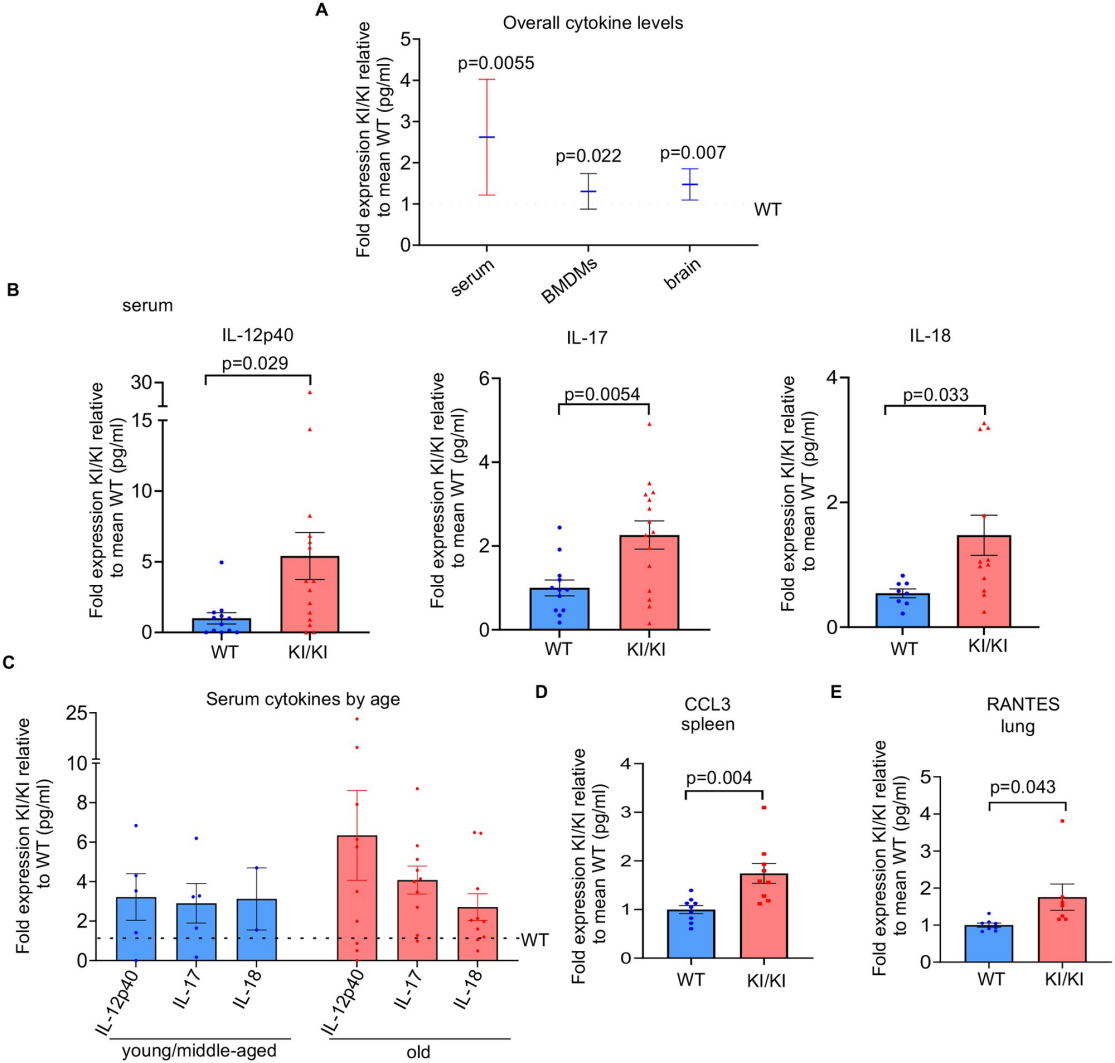
Expression of cytokines and chemokines are increased in *Rap1* knockin serum, BMDMs, and tissues. **(A)** The overall levels of cytokines and chemokines were significantly elevated in serum (n = 12 WT, 15 *Rap1*^KI/KI^ mice), BMDMs (n = 3 per genotype), and brain tissues (n = 7 WT, 11 *Rap1*^KI/KI^ mice) derived from *Rap1*^KI/KI^ relative to WT as determine using multiplex cytokine analysis. P values were determined by a two-way ANOVA. **(B)** Individual cytokines including IL-12p40, IL-17, and IL-18 were elevated in serum from *Rap1*^KI/KI^. Graphs show the fold expression of cytokines derived from *Rap1*^KI/KI^ relative to WT. P values were determined by student’s unpaired t-tests. **(C)** The fold expression of cytokines in *Rap1*^KI/KI^ serum relative to WT serum from young/middle aged and old mice. **(D-E)** Individual cytokines/chemokines CCL3 in the spleen and RANTES in the lung (*n* = 9 mice per genotype) were elevated in *Rap1*^KI/KI^ compared to WT tissues. P values were determined by student’s unpaired *t*-tests. Data are mean ± SEM in all graphs. WT was set to 1 in A-E with KI/KI values displayed as relative to the mean WT value for each graph.

**Fig 5:**
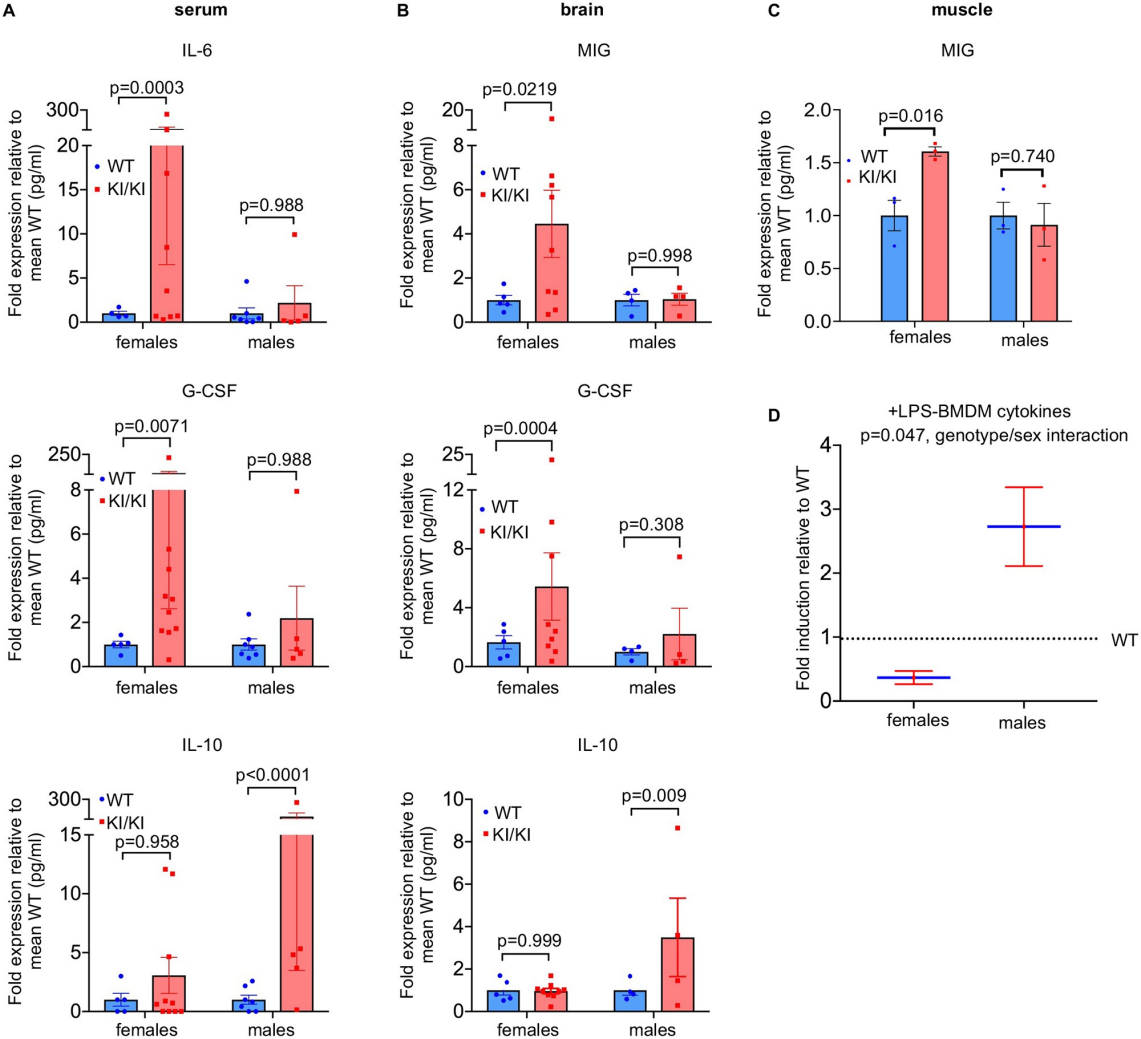
*Rap1* knockin mice exhibit sex-dependent differences in immune responses. **(A)** Bar graphs show the fold expression of cytokines in *Rap1*^KI/KI^ relative to WT serum (*n* = 5 WT females, 10 KI/KI females, 7 WT males, 5 KI/KI males). IL-6 (top) and G-CSF (middle) were significantly increased in *Rap1*^KI/KI^ females only, whereas IL-10 (bottom) was significantly increased in *Rap1*^KI/KI^ males only. P values were determined by two-way ANOVAs with Tukey’s multiple comparisons. **(B)** Bar graphs show the fold expression of cytokines in *Rap1*^KI/KI^ relative to WT brains (n = 5 WT females, 9 KI/KI females, 4 WT males, 4 KI/KI males). MIG (top) and G-CSF (middle) were significantly higher in *Rap1*^KI/KI^ females only, whereas IL-10 (bottom) shows increased expression in *Rap1*^KI/KI^ males only. P values were determined by two-way ANOVAs with Tukey’s multiple comparisons. **(C)** Bar graph shows the fold expression of MIG in *Rap1*^KI/KI^ relative to WT muscles (n = 3 WT females, 3 KI/KI females, 3 WT males, and 3 KI/KI males). MIG was significantly higher in *Rap1*^KI/KI^ females only. **(D)** Graph shows the expression of LPS (200 ng/ml for 24 hr)- induced cytokines/chemokines in BMDMs derived from *Rap1*^KI/KI^ relative to WT females and males. P values were determined by two-way ANOVAs with Sidak’s multiple comparisons. Data are mean ± SEM.

As *Rap1*^KI/KI^ mouse tissues showed elevated immune-response transcripts, we analyzed whether *Rap1*^KI/KI^ mice exhibited perturbation of cytokines and chemokines in serum and various tissues (S4 Table). We found an increase in overall cytokine levels in serum and tissues from *Rap1*^KI/KI^ mice of varying ages, including the pro-inflammatory cytokines IL-12p40, IL-17, and IL-18, CCL3 and RANTES (Fig 4 and S7 Fig and S4 Table). The raw expression values for all cytokines are displayed in S4 Table. Interestingly, some cytokines were upregulated in a sex-dependent manner (Fig 5A–5C). The pro-inflammatory cytokines IL-6 and G-CSF were only upregulated in the serum of *Rap1*^KI/KI^ females, whereas the anti-inflammatory cytokine IL-10 was only upregulated in the serum of *Rap1*^KI/KI^ males (Fig 5A). Similarly, the pro-inflammatory cytokines MIG and G-CSF were upregulated in the brains (Fig 5B) and/or muscles (Fig 5C) of *Rap1*^KI/KI^ females only, and increased IL-10 was observed in the brains of *Rap1*^KI/KI^ males only (Fig 5B).

We further investigated if *Rap1*^KI/KI^ mice exhibited an aberrant response to inflammatory stimuli. BMDMs from WT and *Rap1*^KI/KI^ mice were treated with lipopolysaccharide (LPS) for 24 hours. Compared to untreated BMDMs, most inflammatory cytokines exhibited higher levels in response to LPS (S3 Table). Compared to WT mice, the induction of inflammatory cytokines was markedly lower in BMDMs derived from female *Rap1*^KI/KI^ mice (Fig 5D). In contrast, male *Rap1*^KI/KI^ mice exhibited an overall higher induction of inflammatory cytokines (Fig 5D). Therefore, *Rap1*^KI/KI^ mice may exhibit sex-dependent differences in NF-κB activation and immune responses.

### An increase in spontaneous tumor incidence in mice expressing extratelomeric RAP1

**Fig 6:**
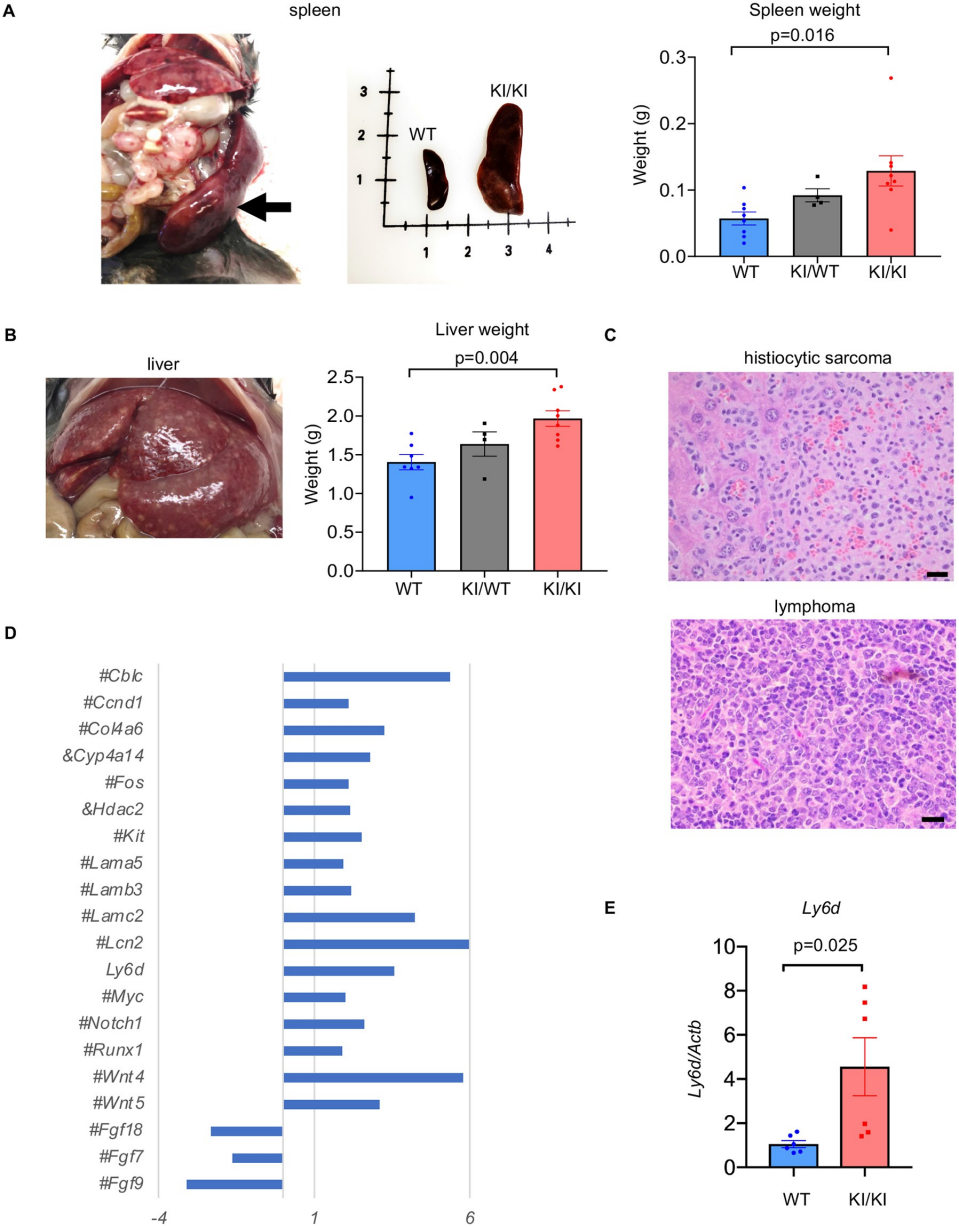
*Rap1* knockin mice have enlarged spleens and livers and higher tumor incidence. **(A)** Far left image shows enlarged spleen that was 3.7 cm long, 12 mm wide, and 3.5 mm thick derived from a *Rap1*^*KI/WT*^ mouse. Also shown is a side-by-side comparison of a spleen derived from a WT and a *Rap1*^KI/KI^ mouse. Bar graph shows weights of spleens from WT (n = 8), *Rap1*^*KI/WT*^ (n = 4), and *Rap1*^KI/KI^ (n = 8) littermates. **(B)** enlarged liver with extensive infiltration of histiocytic sarcoma (left image). Bar graph shows weights of livers from WT (n = 7), *Rap1*^*KI/WT*^ (n = 4), and *Rap1*^KI/KI^ (n = 8) littermates. **(C)** H&E stains showing the presence of histiocytic sarcoma infiltration in the liver of a *Rap1*^KI/KI^ mouse (upper) and lymphoma in the mesenteric lymph node of a *Rap1*^KI/KI^ mouse (lower). Scale bars: 20 μm. **(D)** Z-scores of transcripts implicated in cancer in *Rap1*^KI/KI^, relative to WT mice. # and & indicate transcripts deregulated in females and male only, respectively. **(E)** Levels of *Ly6d* mRNA, as determined by RT-qPCR analysis in the WT and *Rap1*^KI/KI^ livers. n = 6 mice per genotype. P-values were determined using student’s unpaired *t*-tests. All data are mean ± SEM.

Given the notable increase in cytosolic RAP1 in several human tumors [16,27], the significant alterations in transcriptional networks involved in the immune response, consistent upregulation of proinflammatory cytokines, and decreased lifespan in *Rap1* knockin mice, we investigated tumor incidence in *Rap1* knockin compared to WT mice. We monitored spontaneous tumor formation and examined tissues with gross abnormalities or masses by histopathological analysis in WT, *Rap1*^KI/WT^ and *Rap1*^KI/KI^ mice (Fig 6A–6C and S5 Table). Gross abnormalities included enlarged spleens and livers in both female and male *Rap1*^KI/WT^ and *Rap1*^KI/KI^ mice, particularly prominently in *Rap1*^KI/KI^ mice with an increased *Rap1* KI dosage (S5 Table). The weights of whole spleens and livers without obvious anomalies were also increased in *Rap1*^KI/WT^ and *Rap1*^KI/KI^ mice compared to WT controls (Fig 6A and 6B). There was a significant trend toward increased tumor burden with increasing *Rap1* KI dosage from WT to *Rap1*^WT/KI^ to *Rap1*^KI/KI^ and a significant increase in tumor incidence in *Rap1*^KI/KI^ mice, with a prevalence for tumor formation in the liver and spleen (Fig 6C and S5 Table).

Because the liver was a frequent location for tumor formation in *Rap1*^KI/KI^ mice, we determined if transcripts implicated in tumorigenesis were dysregulated in *Rap1*^KI/KI^ liver tissues utilizing gene ontology/pathway and reverse transcription (RT) followed by quantitative (q) PCR analysis. Indeed, 20 transcripts implicated in tumorigenesis were dysregulated in *Rap1*^KI/KI^ compared to WT livers (Fig 6D). Interestingly, *Ly6d* was significantly upregulated in all *Rap1*^KI/KI^ compared to WT mice (S1 Table and Fig 6E). Although there is no evidence to support a causal role for these transcriptional alterations in tumor formation, we note that *Ly6d* mRNA, encoding a cell surface glycoprotein, is upregulated in various types of tumors in humans [28] as well as liver tumors in mice [29].

### Mice expressing extratelomeric RAP1 exhibit metabolic dysfunction

Obesity and metabolic dysfunction are associated with an increased risk of cancers [30]. Previous reports have demonstrated obesity and metabolic dysfunction in *Rap1* null mice [6,8]. The *Rap1*^KI/KI^ mice provided an opportunity to determine if these phenotypes were due to complete RAP1 loss and/or loss of telomeric RAP1. Similar to *Rap1* null mice, *Rap1*^KI/KI^ females exhibited increased body weight compared to WT females (S8A and S8B Fig). The increased body weight of *Rap1*^KI/KI^ females was not due to increased food intake in these mice (S8C and S8D Fig). These results are in agreement with transcriptional alterations of genes involved in metabolism in *Rap1* KI mice.

To further assess metabolic function, mice were challenged with a high-fat/high-sugar diet (HFHS). On the HFHS diet, *Rap1*^KI/KI^ females showed accelerated body weight and higher fasting blood glucose levels compared to WT females (S8E–S8H Fig). To determine if extratelomeric RAP1 affected metabolism in response to the HFHS diet, we examined body weight and fasting blood glucose of *Rap1*^KI/WT^ mice that had increased cytosolic RAP1, but similar RAP1 protein levels in comparison to WT (Fig 1F and 1G). Compared to WT females, *Rap1*^KI/WT^ females on the HFHS diet exhibited a trend for higher body weight and fasting blood glucose levels (S8E and S8G Fig), indicating that increased extratelomeric RAP1 exacerbated metabolic dysfunction in response to metabolic challenge. On the other hand, male *Rap1*^KI/KI^ and *Rap1*^KI/WT^ mice did not show significant differences in body weights or fasting blood glucose levels (S8B, S8F and S8H Fig). Previous findings in *Rap1* null mice also reported a female-specific body weight gain and overall defect in glucose metabolism [6,8].

### Mice expressing extratelomeric RAP1 display both sex-dependent and sex-independent neurological deficits

**Fig 7:**
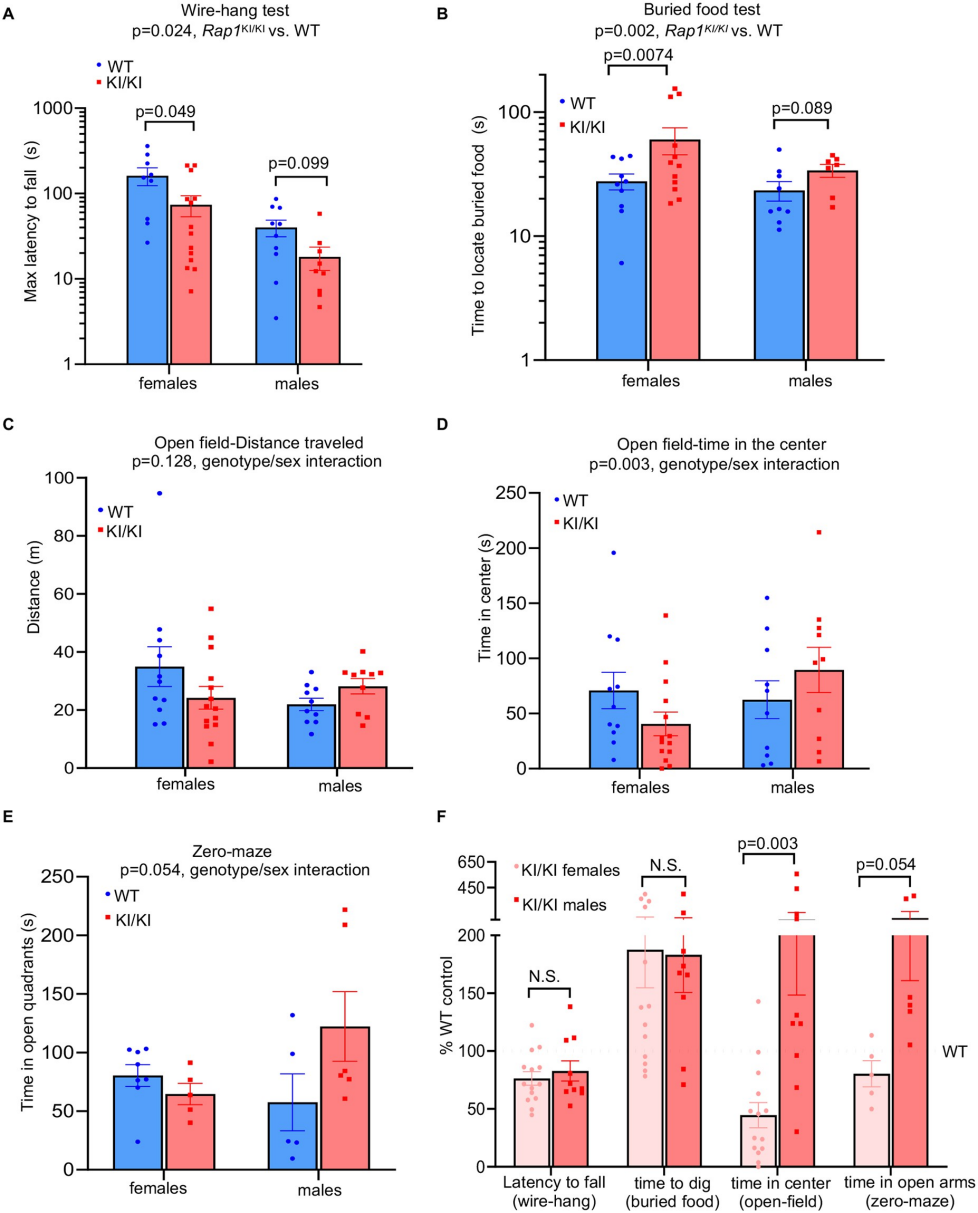
*Rap1* knockin mice exhibit neurological changes in a sex-dependent manner. **(A)** Wire-hang test showing maximum latency of mice to fall, *n = 19* WT (9 females and 10 males) and *n = 24 Rap1*^KI/KI^ (14 females and 10 males) mice. P values in WT vs. *Rap1*^KI/KI^ females and /or males were shown. **(B)** buried food test. *n = 19* WT (10 females and 9 males), *n = 19 Rap1*^KI/KI^ (12 females and 7 males). P values in WT vs. *Rap1*^KI/KI^ females and/or males were shown. **(C)** open-field test/distance traveled in 15 mins. *n* = 21 WT (11 females and 10 males) and *n = 24 Rap1*^KI/KI^ (14 females and 10 males). *p* = 0.128 (WT vs. *Rap1*^KI/KI^). **(D)** open-field test/time in the center out of 15 mins. *p* = 0.003 (genotype/sex interaction). **(E)** zero-maze test, *n = 14* WT (8 females and 5 males) and *n = 11 Rap1*^KI/KI^ (5 females and 6 males) mice. *p* = 0.054 (genotype/sex interaction). **(F)** Data for behavioral tests displayed as % WT control (set to 100%). Behavior results were analyzed in R software with 2-way ANOVA using genotype and sex as factors, with covariates of wave and body weight included where appropriate. All data are mean ± SEM.

Telomerase null mice with critically short telomeres exhibit deficits in learning and memory, neuromuscular coordination, and olfaction [31,32]. However, the mechanisms by which telomere impairment promotes these deficits remain elusive. *Rap1* knockin mice showed dysregulation in a high number of immune-response transcripts/pathways and pro-inflammatory cytokines in brain and muscle tissues, which could promote chronic inflammation that disrupts neurotransmission and neuroplasticity. Thus, we performed behavioral tests to assess motor, sensory, affective, and cognitive functions. *Rap1*^KI/KI^ mice displayed shorter latencies to fall from an inverted wire grid (Fig 7A), which were significant with or without statistical correction for body weight. This suggests reduced limb strength and/or coordination. Additionally, fasted *Rap1*^KI/KI^ mice were slower to locate a buried food pellet (Fig 7B), indicating impairment in olfactory sensitivity. Because both of these deficiencies develop with normal aging in mice [33,34], these results may suggest accelerated neurological aging in *Rap1*^KI/KI^ mice, which was more pronounced in females. Exploratory locomotion did not differ in sex-genotype interaction in the open field tests (Fig 7C), but the influence of the *Rap1* knockin mutant on time in the center, an indication of anxiety, differed significantly between females and males (Fig 7D). Genotype-by-sex interactions were observed in the time spent in the open quadrants of a zero maze (Fig 7E) and a non-significant trend was observed for the light-dark box test (S9A Fig), which are also measures of anxiety-like behavior [35]. In all tests, the direction of effects was for decreased and increased anxiety-like behavior in *Rap1*^KI/K^ males and females, respectively. Interestingly, human studies have demonstrated a positive correlation between anxiety and short telomeres [36,37], although those associations were not sex dependent. Finally, to evaluate cognitive abilities of *Rap1*^KI/KI^ mice, we assessed working memory in a spontaneous alternation task (Y-maze) and cognitive flexibility in a reversal learning task (Water T-maze). No significant differences were observed in either test (S9B and S9C Fig). In sum, we found impairments in motor and olfactory function in male and female *Rap1*^KI/KI^ mice, whereas anxiety was altered bidirectionally in a sex dependent manner (Fig 7F) and cognitive function remained intact.

### Neuroinflammation is associated with astrogliosis and upregulation of inflammasome pathway components in the *Rap1* knockin mouse brain

**Fig 8:**
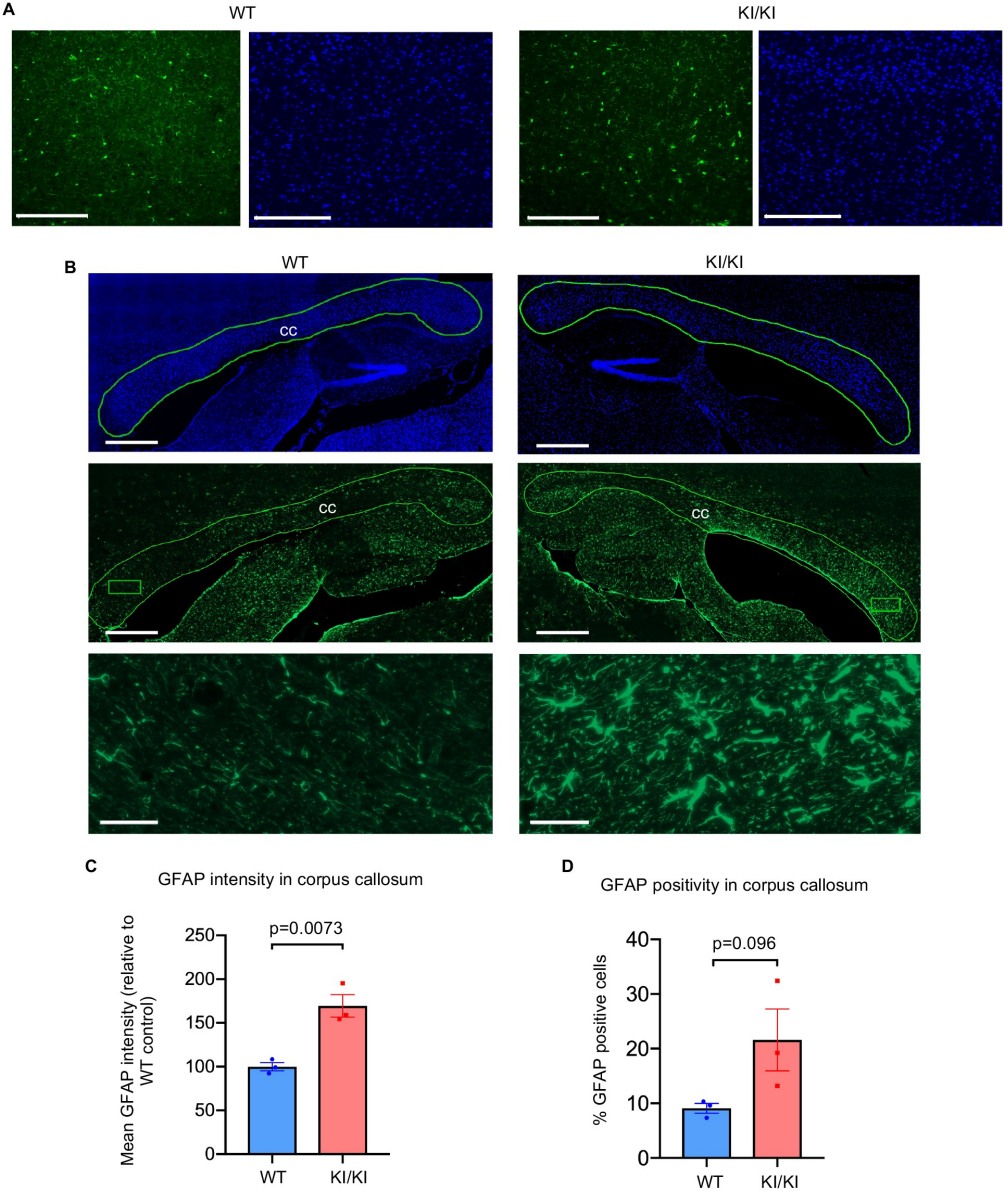
*Rap1* knockin mice exhibit astrogliosis in the corpus callosum. **(A)** Representative images from sagittal brain sections derived from WT and *Rap1*^KI/KI^ mice showing **(A)** the cerebral cortex stained with anti-IBA1 (green) and mounted with medium containing DAPI (blue). Scale bars: 200μm. **(B)** Representative sagittal brain sections were mounted with medium containing DAPI (top panel, blue) and stained with anti-GFAP (middle panel, green). Top and middle panels display regions containing the corpus callosum (outlined in green). Boxed regions within the corpus callosums are shown at a higher magnification in the bottom. Scale bars: 500 μm (top and middle panels); 40 μm (bottom panel). **(C-D)** Quantification of the mean intensity of GFAP staining and percentage of GFAP-positive cells within the corpus callosum (outlined region). *n* = 3 mice per genotype, Data are mean± SEM. P values were obtained using student’s unpaired *t*-tests.

To investigate the source of low-grade neuroinflammation and/or origin of the observed behavioral deficits in the *Rap1*^KI/KI^ brain, we performed immunostaining on sagittal brain sections derived from WT and *Rap1*^KI/KI^ mice. The major effectors of neuroinflammation in the brain are glial cells, including astrocytes and microglia (brain resident macrophages) [38,39]. To identify astrocytes and microglia and examine differences in their numbers, morphology, and staining intensity, we utilized antibodies that recognize glial fibrillary acidic protein (GFAP) and ionized calcium-binding adaptor molecule 1 (IBA1), respectively. Immunostaining for IBA1 revealed no differences in the characteristics of microglia in *Rap1*^KI/KI^ compared to WT brains (Fig 8A); however, GFAP immunostaining revealed a significant increase in GFAP staining intensity (Fig 8B and 8C) and a trend towards an increase in the number of GFAP-positive astrocytes in the corpus callosum (Fig 8B and 8D). Astrocytes in the corpus callosum exhibited an enlarged morphology (Fig 8B). These findings indicate the presence of reactive astrocytes (astrogliosis) in the corpus callosum, which are known to secrete inflammatory cytokines and drive neuroinflammation [38,39]. Consistent with these findings, we observed a significant increase in inflammasome pathway components, including cleaved caspase-1, pro-IL-1β, IL-18 in *Rap1* knockin brain tissues, which was more pronounced in females (S10 Fig). These data suggest that reactive astrocytes and inflammasome activation may contribute to the increased expression of inflammatory cytokines in *Rap1*^KI/KI^ brain tissues.

## Discussion

Telomere attrition is a hallmark of aging [2] and is associated with age-related pathologies, including cancer, diabetes mellitus, and neurodegenerative disorders [40]. A remaining question is the degree to which these phenotypes are driven by a telomere-induced DNA damage response versus other emergent properties of telomeric proteins that occur upon telomere erosion. RAP1 is an important case in point; in budding yeast, telomere attrition leads to localization of RAP1 away from telomeres and contributes to gene expression changes that occur during senescence [19,20]. Furthermore, loss of RAP1 in mice leads to profound alterations in the immune response and metabolism, and the extent to which this is triggered by loss of RAP1 from telomeres or its other roles in controlling gene transcription is not yet understood [6,8,10,14]. To understand the impact of aberrant RAP expression and cellular localization in life- and health- span, we generated and characterized a *Rap1* knockin mouse model. By disrupting the RAP1-TRF2 interaction, we eliminated RAP1 localization at the telomere, which led to its extratelomeric localization, including in the cytoplasm. Similar to deletion of RAP1 alone [7,9], removal of RAP1 at telomeres did not induce telomere erosion or a telomeric DDR. Thus, we could address the extent to which these other properties of RAP1 contribute to pathophysiology in mammals. We found that *Rap1* knockin mice, although viable, had a reduced lifespan and several other defects in immune function, metabolism, and behavior. Thus, our studies support the notion that alterations in RAP1 expression and localization contribute to pathophysiologies in a telomere-induced DDR-independent manner.

Previous studies have demonstrated that RAP1 levels are dependent on its interaction with TRF2 in mice [7,41]. Thus, the inability of RAP1 I312R to bind TRF2 may have contributed to the decreased RAP1 protein expression in *Rap1*^KI/KI^ mice. The increased cytosolic/decreased nuclear distribution of RAP1 in the *Rap1*^KI/KI^ mice provides evidence that the RAP1-TRF2 interaction helps facilitate RAP1 nuclear retention. Notably, changes in RAP1 were independent of TRF2 expression or localization, which was unaltered in *Rap1*^KI/KI^ mice.

Non-telomeric RAP1 can regulate gene expression [6,14–16]. Thus, altered RAP1 levels and/or a switch from telomeric to non-telomeric localization in RAP1 may affect gene transcription and contribute to pathological changes in *Rap1* KI mice. Indeed, our data revealed a large number of transcripts (Fig 3 and S5 Fig and S1 Table) and pathways (S2 Table) that were altered in various *Rap1* KI mouse tissues, especially the liver. Downregulation of metabolic transcripts and pathways in the liver of *Rap1*^KI/KI^ mice is in agreement with the increased body weight and fasting blood glucose observed in these mice, which was more pronounced in female *Rap1*^KI/KI^ mice. These sex-dependent differences in metabolism are in accord with previous findings in *Rap1* null mice [6,8]. This overlapping phenotype and some shared changes in metabolic pathways, including downregulation of the PPARα pathway in *Rap1*^KI/KI^ (S2 Table) and *Rap1* null livers [8], suggest that the increased body weight and metabolic defects in *Rap1* KI mice may be due to the decreased expression of RAP1 in *Rap1* KI mice. However, *Rap1*^KI/WT^ mice had RAP1 levels comparable to those of WT mice (Fig 1F), yet showed a trend for higher body weight and fasting blood glucose levels with a HFHS diet (S8E and S8G Fig). Therefore, it seems feasible that metabolic dysfunction in *Rap1*^KI/KI^ mice may also be augmented by extratelomeric RAP1.

In addition to transcriptional perturbations, cytosolic RAP1 colocalizes with the NLRP3 inflammasome in neutrophils [42] and activates NF-kB [16]. These cytosolic factors play fundamental roles in cytosolic inflammation and immune responses [18,43]. Although *Rap1* deletion results in defective NF-kB activation [16], we found that *Rap1*^KI/KI^ brain and/or bone marrow displayed signs of NF-kB and/or inflammasome activation. Pro-inflammatory cytokine production was increased in *Rap1*^KI/KI^ brain tissues, bone marrow derived macrophages, and serum. The increases in immune response transcripts and pathways were observed in all tissues examined. These findings are in agreement with reports by Teo and Liu et al [16,42] and suggest that increased cytosolic RAP1 may promote NF-kB and inflammasome activation that, in turn, drives increased immune response transcripts and pro-inflammatory cytokine production in *Rap1*^KI/KI^ mice. Interestingly, we observed sex differences in the immune response and basal pro-inflammatory and anti-inflammatory cytokine levels in *Rap1*^KI/KI^ mice. We speculate that sex differences in genomes and/or proteomes facilitate divergent interactions with extratelomeric RAP1, leading to sex-dependent deregulation of downstream pathways that affect cytokine production. Consequently, sexual dimorphism in pro- or anti-inflammatory basal cytokine production in *Rap1*^KI/KI^ BMDMs could explain the observed sex differences in their ability to mount an effective pro- or anti-inflammatory response to inflammation stimuli. Similar sex/gender dimorphism in LPS-induced pro-inflammatory cytokines has been observed in humans and rodents [44]. Thus, it would be of great interest to investigate the potential role for RAP1 in regulating sex-dependent immune responses and disease susceptibility in humans.

Increased cytosolic RAP1 has been reported in several human tumors, including breast cancer and non-small cell lung cancer [16,27]. In addition, *Rap1* deficiency accelerates tumor incidence in mice, in response to carcinogens and the Myc oncogene [45,46]. It is reasonable to deduce that decreased levels of RAP1 and/or elevated RAP1 cytosolic distribution may have promoted the elevated tumor incidence in *Rap1*^KI/KI^ mice. Lymphomas and histiocytic sarcomas were the predominant tumors observed in *Rap1*^KI/KI^ mice, primarily detected in the livers and spleens. The weights of these two organs were also increased. Therefore, the mouse liver and spleen may be the most susceptible to tumorigenesis in response to changes in RAP1. Thus, significant alterations in transcriptional networks implicated in tumorigenesis and the immune response as well as the upregulation of proinflammatory cytokines may have contributed to the increased tumor burden and accelerated mortality in *Rap1*^KI/KI^ mice.

Although *Rap1* knockin mice share some pathological changes with *Rap1* null mice, e.g. increased body weight, they are distinct from *Rap1* null mice in which both telomeric and non-telomeric RAP1 are eliminated. Less than 5% of deregulated genes overlapped in *Rap1* knockin and null liver tissues [6,8] (S11 Fig). Since the primary difference between *Rap1* knockin mice with WT or *Rap1* null mice is the presence of RAP1 distal to telomeres with altered cytosolic and nuclear distributions, we posit that these changes in RAP1 could modify the transcriptional landscape in a manner that could not be achieved via the absence of RAP1 alone. Of the overlapping deregulated genes between *Rap1*^KI/KI^ and null livers, more than half were regulated inversely (S11 Fig). Other pathways in *Rap1*^KI/KI^ mice were also regulated inversely to those regulated in *Rap1* null mice. For example, pathways implicated in energy metabolism, including oxidative phosphorylation and electron transport pathways were downregulated in *Rap1*^KI/KI^ livers, but upregulated in *Rap1* null livers [8]. *Rap1* knockin mice had decreased lifespans, which was not observed in *Rap1* null mice [8]. In addition, *Rap1* knockin mice showed increased spontaneous tumor incidence and altered behaviors, which were not reported in *Rap1* null mice in the absence of any cancer-prone genetic background or carcinogen exposure [45,46]. Although the decreased lifespan in *Rap1* knockin mice suggests a dominant negative effect of the KI allele, we posit that this phenotype is rather a consequence of increased extratelomeric/cytosolic RAP1. This notion is supported by our finding that *Rap1* knockin mice exhibited increased cytosolic RAP1 and increased NF-κB activation/expression of pro-inflammatory cytokines. In contrast, deletion of *Rap1* leads to defective NF-κB activation and pro-inflammatory cytokine production [16,47,48]. These observations support the notion that the KI allele behaves as wild type. Thus, *Rap1* knockin mice present an opportunity to study the role of TRF2-independent RAP1 functions distal to telomeres and their role in aging and age-related phenotypes.

*Rap1*^KI/KI^ mice displayed deficits in behavioral tests of locomotor and chemosensory function. Decline in these functions are well-documented during the normal aging process of mice and humans alike [49,50]. Immune-response transcripts were most upregulated in the *Rap1*^KI/KI^ brain, in agreement with increased pro-inflammatory cytokine production in this tissue. Immunostaining revealed astrocytes within the corpus callosum as a potential source of the neuroinflammation. Because experimental damage to the corpus callosum causes impairment in tests of motor coordination [51,52], it is possible that astrogliosis in the corpus callosum may have contributed to some of the deficits exhibited by the *Rap1*^KI/KI^ mice. *Rap1*^KI/KI^ mice displayed deficits in the wire-hang and buried-food test. It is noteworthy that mice with normal aging or neurodegenerative diseases display white matter injury and hypoplasia in the corpus callosum, deficits in the wire-hang test, and/or olfactory dysfunction [53–58]. Olfactory dysfunction also increases with telomere shortening [31]. Collectively, these observations suggest that aberrations in RAP1 may contribute to age-related decline in locomotor and sensory function and accelerate certain aspects of normal aging. Interestingly, *Rap1*^KI/KI^ mice showed behavioral changes that were sexually dimorphic. *Rap1*^KI/K^ mice female mice exhibited increased anxiety like behavior, whereas *Rap1*^KI/KI^ male mice displayed decreased anxiety like behavior. These phenotypic features are consistent with data in humans, where approximately 33.7% of the population experiences an anxiety disorder during their lifetime, and with an almost doubling of the prevalence of anxiety related disorders in women as in men [59]. The fact that aberrant RAP1 heightens anxiety in females and lowers anxiety in males warrants further investigation as to whether RAP1 may contribute to sex-dependent differences in anxiety.

Previous studies in humans have shown that RAP1 protein levels decrease with aging [23]. We found that the inability of RAP1 I312R to bind TRF2 leads to decreased RAP1 levels and increased cytosolic/decreased nuclear distribution of RAP1 in mice, a trend that was also observed in human cells undergoing gradual telomere attrition. We hypothesize that telomere shortening over time may alter the expression and localization of RAP1 and thereby contribute to age-related pathologies. Our data establish in a physiologically relevant setting that aberrant expression/localization of RAP1 can lead to age-associated and sex-specific properties distinct from its role at the telomere itself. Our findings provide important additional evidence that the re-localization of RAP1 away from telomeres may contribute to aging phenotypes in mice in a manner that is separable from the induction of a telomeric DDR [20,60]. Our studies support the notion that the loss of telomere function, or in this case the loss of the telomere-binding activity of RAP1, can contribute to emergent properties that were not triggered by telomere damage *per se*. Our findings further establish that, despite an ability of non-telomeric RAP1 to largely mimic the gene expression profiles of wild-type MEFs [6], there is a sufficient perturbation in expression of cytokine and immune responsive genes to result in marked sex-specific phenotypes in mice, and decreased lifespan. Our data suggest that mechanisms underlying age-associated disease and telomere biology disorders are multifaceted and that the telomeric protein RAP1 plays crucial pleiotropic roles in tissue homeostasis and organismal aging.

## Materials and methods

### Ethics statement

All animal experiments were carried out according to the “Guide for the Care and Use of Laboratory Animals” (National Academy Press, USA, 1996), and were approved by the Institutional Animal Care and Use Committee of National Institute on Aging (ASP #383-LGG-2025).

### Mice and cell cultures

The *Rap1* I312R knockin mice were generated using Crispr/Cas9 technology [61]. Briefly, a sgRNA (GAACTGCCATTAAGGTGATC) and a single-strand donor oligonucleotides was generated by IDT (https://www.idtdna.com/pages), which contains the desired mutation (CAGCCAGACGAGGAGGAAGAAGAACCAAAAGTTTCTACGCAAGAAGTGGGAACTGCCATTAAGGTG**CGG**CG**C**CAGCTAATGGAGAAGTTCAACTTGGATCTATCAACAGTTACACAGGCC; the four bold and underlined nucleotides were changed from the wildtype sequence for introducing the true and silent mutations). The *Rap1* I312R knockin mice were backcrossed to C57BL/6N strain for four times. *RelA*-EGFP reporter mice were described previously [26]. Lifespan was determined using the Kaplan-Meier survival analysis. Mice died naturally (the animals were found dead in the cage) or euthanized prior to any noticeable distress according to NIA-ACUC Policy 005 humane endpoint criteria, as described alike [49,50]. Following euthanasia, whole bodies or organs with gross abnormalities and masses were submitted to the NIH Division of Veterinary Resource for histopathological analysis.

Primary MEFs were isolated from 13.5-day embryos. Primary MEFs and human fibroblasts were cultured in DMEM supplemented with 10–20% FBS, 2 mM L-glutamine, 100 U/ml penicillin, and 100 μg/ml streptomycin. BMDMs were obtained by culturing mouse bone marrow in 15% FBS, 2 mM L-glutamine, 100 U/ml penicillin, 100 μg/ml streptomycin, 1.5% 1 M Hepes, 1% non-essential amino acid solution (#11140050, ThermoFisher Scientific), and 15% conditioned medium from L929 cells, containing granulocyte macrophage-colony stimulating factor (GM-CSF).

### Mouse weight, food intake, fasting blood glucose analysis

Mice fed a standard chow diet (ad lib) were weighed weekly between the ages of 14 and 74 weeks. Mice fed a high fat high sugar diet (ad lib) consisting of 20.3% protein, 47% carbohydrates, 27.1% fat, 11.5% sucrose, and 31% dextrose by weight (#103806, Dyets, Bethlehem, PA) were weighed weekly between 6 and 20 weeks of age. To measure food intake, 50g of chow per mouse was placed in each cage. One week following food distribution, the remaining food in the cages was weighed. To measure fasting blood glucose, mice were fasted overnight (~17 hr). Blood was drawn from the mouse tail veins and placed directly onto Accu-chek blood glucose strips (# K6570210410, ADW Diabetes, Pompano Beach, FL), and measured for fasting blood glucose readings by an Accu-chek glucose monitor (# C6570210110, ADW Diabetes).

### Mouse behavioral tests

Mouse behavioral assessments were performed on mice at middle age (12–17 months), with ages matched across sex and genotype (*F*_*3*,*40*_ = 1.08, *p*>0.5). Testing was repeated on two separate occasions with approximately equal representation of groups. Animal movement in chambers and water mazes was tracked with digital cameras and ANY-Maze software (Stoelting Co; Wood Dale, IL). *Wire hang test*: mice were tested for latency to fall from an inverted wire cage top. Three trials were conducted, spaced by 30 minutes. *Open field*: mice were recorded for 15 minutes in a 40 x 40 x 40 cm opaque chamber. *Spontaneous alternation*: mice were recorded for 8 minutes in a symmetrical Y-maze apparatus. Percentage of arm entries that were alternations (three successive entries into distinct arms) were calculated using the formula (# alternations) / (# arm entries– 2). *Reversal learning*: procedures were adapted from Filali et al [62]. Mice were first trained to find a hidden platform located in one arm of a T-maze submerged in water colored with non-toxic paint. For the initial phase, the platform was always located in the same arm for any given mouse. After reaching criterion of 10 consecutive correct trials (reaching platform without entering the incorrect arm), the platform was moved to the opposite arm and each animal was trained to a new criterion of 5 consecutive correct trials. *Light-dark box*: mice were tested for 10 minutes in an apparatus containing dark (black walls and ceiling) and brightly-lit (750 lux) compartments, connected by a small 5.5 x 5.5 cm opening. Transitions between compartments were recorded. Buried food test: mice were fasted overnight and tested for latency to find a 45 mg food pellet buried under 3 cm of bedding. Latency to dig continuously for at least 2 seconds above the location of the buried pellet was recorded. To verify food motivation, mice were subsequently presented with an identical pellet placed on top of bedding [63]. Five mice did not eat the exposed pellet within one minute and were thus excluded from analysis in this test.

### Cytokines and NF-κB activation

Cytokine/chemokine levels, except IL-18 levels, were quantified using a mouse cytokine/chemokine 31-plex array (# MD31) by Eve Technologies (Calgary, AB Canada). IL-18 was measured using an ELISA kit (MBL international, #7625). IL-1β was measured using an ELISA kit (R&D Systems, #MLB00C) on a subset of WT, *Rap1*^KI/WT^, and *Rap1*^KI/KI^ brain homogenates. To measure NF-κB activation, cells were treated with LPS derived from Escherichia coli (200ng/ml, O111:B4, Sigma-Aldrich) for 24 hr. Nuclei were isolated using a nuclear extraction kit (# ab113474, Abcam), and the levels of p65 in the nuclei were determined using the NF-κB p65 transcription factor assay kit (# ab133112, Abcam). To measure GFP positive cells and GFP-signal intensity in *RelA*-EGFP mice, single cells obtained from bone marrow and spleen were analyzed on a BD FACS Canto II Flow Cytometer (Becton Dickinson, Franklin Lakes, NJ). Results were analyzed using FlowJo software (Becton Dickinson).

### Telomere length, telomere dysfunction-induced foci, and SA-beta gal analysis

Telomere length was determined by quantitative telomere in situ hybridization (Q-FISH) [64] and telomere restriction fragment analysis [65] using a Cy3-labeled (CCCTAA)_3_ PNA probe (Panagene) and a Telo-C-Biotin (CCCTAA)_3_ probe (PNA Innovations), respectively. TIFs were detected by immunofluorescence and telomere-FISH using primary γH2AX antibody (#05-636-AF488, Millipore) and Alexa Fluor 488 dye-conjugated secondary antibody (#R37120, Invitrogen), followed by fixation and telomere-FISH [65]. TIFs were scored by the co-localization of γ-H2AX and telomere-FISH signal. β-galactosidase activity was carried out using the SPiDER-β-galactosidase staining kit (#SG04-1, Dojindo).

### Protein detection

ELISAs were used to determine NF-κB and cytokine expression. Immunofluorescence was used to determine IBA1 and GFAP expression using anti-IBA1 (Abcam, ab 178846) and anti-GFAP antibody (Abcam, ab 7260), respectively. Western blotting was employed to determine expression of RAP1 (Santa Cruz, sc-53434), TRF2 (Novus Biologicals, NB110-57130), GAPDH (ABclonal, AC027), β-ACTIN (Cell Signaling Technology, 497OS), α-TUBULIN (Millipore Sigma, T5168), LAMIN A (Abcam, ab26300), H3 (Abcam, ab1791), and inflammasome components [pro-CASPASE-1/cleaved CASPASE-1 (Adipogen, AG AG-20B-0042-C100), NEK7 (Santa Cruz, sc-393539), pro-IL-18 (Genetex, GTX32675), IL-18 (MBL International, D046-3), pro-IL-1β (Cell Signaling Technology, 12242)]. For cell fractionation, cells were incubated with buffer A [(hypotonic, 10mM Hepes pH 7.9, 10mM KCl, 0.1mM EDTA, 0.1mM EGTA, and Halt protease inhibitor cocktail (ThermoFisher Scientific)] for 15 minutes on ice, treated with ice cold 10% IGEPAL CA-630 (Sigma-Aldrich), and vortexed for 10 seconds to burst the swollen cytoplasmic membranes. Cells were centrifuged briefly (~10 seconds). After collecting supernatant containing cytoplasmic factions, the nuclear pellet was resuspended in ice-cold buffer C (20 mM Hepes pH 7.9, 400 mM NaCl, 1 mM EDTA, 1 mM EGTA, and Halt protease inhibitor cocktail) for 15 minutes with rocking at 4°C, and then spun down at 5,000xg. Supernatant was collected for nuclear fractions. Equal amounts of cytoplasmic and nuclear fractions were used for western blotting.

### Detection of mRNA

Microarray procedures and data analyses were conducted at the Gene Expression and Genomics Unit at the National Institute on Aging (Baltimore, MD), as described [66]. Brain and liver tissues were homogenized and dissolved in 1 mL cold RLT buffer containing 10% β-mercaptoethanol (BME) together with glass beads. Heart and muscle tissues were dissolved in 1mL cold RLT buffer +10% BME with zirconia beads. Tissues were homogenized with beads using a Precellys 24 Tissue homogenizer (Bertin Instruments, Rockville, MD) at 5500–6000 rpm for 30 seconds, and centrifuged at 12,000 rpm for 4 min at 4°C to remove undissolved tissues and beads. RNA was extracted using RNeasy mini kits (# 74104, Qiagen, Hilden, Germany) for brain and liver tissues and using RNeasy fibrous tissue mini kit (# 74704, Qiagen) for heart and muscle tissues.

Quantitative reverse transcriptase polymerase chain reaction (RT-qPCR) was used to determine and validate the expression of altered transcripts in *Rap1*^KI/KI^ mice. Total RNA was converted to cDNA using the high-capacity cDNA reverse transcription kit (# 4368814, ThermoFisher Scientific). Amplification of cDNA was performed using SYBR green PCR mastermix (# 4364344, ThermoFisher Scientific) and primers (Eurofins Genomics). Primers sequences are as follows:

*Ly6d* forward: 5’-CAAAACCGTCACCTCAGTGGAG-3’
*Ly6d* reverse: 5’-AGCCATAACAGTGAGCAGGC-3’
*Actb* forward: 5’-GCTTCTAGGCGGACTGTTACTGA-3’
*Actb* reverse: 5’-GCGCAAGTTAGGTTTTGTCAAA-3’
*Gapdh* forward: 5’-ATGTGTCCGTCGTGGATCTGA-3’
*Gapdh* reverse: 5’-CCTGCTTCACCACCTTCTTGA-3’

All reactions were performed in triplicate, and quantitative PCRs were performed using the Bio-Rad CFX Connect Real-Time System. The Δ*C*_t_ values were determined by normalizing gene expression to beta actin (*Actb*) or glyceraldehyde 3-phosphate dehydrogenase (*Gapdh*). To calculate fold differences in mRNA expression, the equation [2^(-ΔΔCt)^] was used.

### Statistical analysis

GraphPad Prism 8.0 (GraphPad Software, Inc.) was used for statistical analyses. The data are expressed as the mean ± standard error of the mean (SEM) or standard deviation (SD). *P* values of *<* 0.05 denoted significant differences. Gehan-Breslow-Wilcoxon tests were used to determine differences in Kaplan-meier survival curves for lifespan analysis. For the tumor incidence data in S5 Table, a Fisher’s exact test and a Cochran-Armitage test (chi-square test for trend) were used for the comparison in tumor incidence in WT and *Rap1* knockin *mice*.

## Supporting information

S1 Fig *Rap1* I312R does not affect protein and mRNA expression of TRF2. **(A-B)** *Rap1* and *Trf2* mRNA expression levels in WT and *Rap1*^KI/KI^ primary MEFs by RT-qPCR. *n* = 5 primary MEF cell lines for each genotype with technical duplicates. (C) Western blot on whole-cell lysates from WT, *Rap1*^*KI/WT*^, and *Rap1*^KI/KI^ primary MEFs. Quantification shows no significant differences in TRF2 protein levels among the genotypes. P values were not significant (N.S.) and determined by a one-way ANOVA with student’s unpaired *t*-tests (**A-B**) and Tukey’s post-hoc comparisons (**C**). Data are mean ± SEM. *n* = 5 primary MEF cell lines for each genotype.
(TIF)

S2 Fig RAP1 levels and cytosolic distribution in primary human fibroblasts upon prolonged culture. **(A)** Representative western blot analysis of RAP1 and TRF2 levels using whole-cell lysates derived from primary BJ fibroblasts at early (E) and late (L) passages (non-dividing). **(B-C)** Representative western blot analysis of RAP1 and TRF2 levels using cytosol (C) and nuclear (N) fractions derived from BJ fibroblasts at early (E) and late (L) passages. Lamin A and tubulin are nuclear and cytosolic proteins, respectively. The percent distribution of RAP1 and TRF2 in each fraction is shown. **(D)** Telomere length measurement of HeLa, HeLa 1.2.11, early passage (E), and late passage (L) BJ fibroblasts by telomere restriction fragment analysis using a biotin conjugated telomere probe.
(TIF)

S3 Fig Telomere dysfunction was not detected in *Rap1* knockin mice. **(A)** Representative metaphase spreads of Q-FISH analysis on WT and *Rap1*^KI/KI^ primary MEFs showing DAPI (grey) and telomere signals (red). Quantitative measurement of telomere signal intensities is shown (right panel). Bars represent mean ± SD. Scale bars: 20μm. **(B-C)** Representative images of TIF analysis by IF-telomere FISH on primary MEFs (B) and mouse spleen tissues (C) using telomere FISH (red) and anti-γH2AX (green). *n* = 172 WT MEFs, *n* = 134 *Rap1*^KI/KI^ MEFs, *n* = ~300 splenocytes per mouse analyzed. WT and *Rap1*^KI/KI^ mice are 20, 22, and 24 months. Cells with ≥3 TIFs were not observed. P values between WT and *Rap1*^KI/KI^ were not significant. Data are mean ± SEM. Scale bars: 5μm.
(TIF)

S4 Fig Cellular senescence was not detected in *Rap1* knockin mice. Representative images (left) of primary WT and *Rap1*^KI/KI^ MEFs stained with SPiDER-β-gal (green) and DAPI (blue). A minimum of 8 images containing ~300 cells were captured. The right panel shows plotted data points for individual images, and each data point was derived from an image. MEFs were at passage 5. Scale bars: 50μm. Data are mean ± SEM.
(TIF)

S5 Fig Gene transcription and ontology are altered in *Rap1* knockin female mice. Gene ontology classifications for the significantly altered transcripts as determined by microarray analysis in the liver **(A)**, brains **(B)**, hearts **(C)**, and skeletal muscles **(D)** of *Rap1*^KI/KI^ compared to WT mice. *n* = 3 mice per genotype.
(TIF)

S6 Fig *Rap1* knockin bone marrow, spleen, and BMDMs show mild NF-κB activation *in vivo*. **(A)** Histograms represent the population of GFP-positive cells (GFP+) in bone marrow (left) and splenocytes (right) derived from WT (blue histograms) or *Rap1*^KI/KI^ (red histograms) mice in *RelA*-EGFP reporter background by flow cytometry analysis. Gating for GFP+ cells is indicated by the dotted line. *n* = 5 WT and *n* = 3 *Rap1*^KI/KI^ mice. **(B)** Bar graph shows NF-κB activation as determined by the level of p65 expressed in nuclear extracts derived from WT and *Rap1*^KI/KI^ mouse BMDMs using the NF-κB p65 transcription factor assay kit. P values were determined using student’s unpaired *t*-tests. All data are mean ± SEM.
(TIF)

S7 Fig Expression of cytokines and chemokines in female and male *Rap1* knockin serum, BMDMs, and tissues. **(A)** The overall levels of cytokines and chemokines were significantly elevated in serum (n = 5 WT female, 10 *Rap1*^KI/KI^ females, 7 WT males, and 5 *Rap1*^KI/KI^ male mice), BMDMs (n = 2 WT females, 2 *Rap1*^KI/KI^ females, and 1 male per genotype), and brain tissues (n = 5 WT female, 9 female *Rap1*^KI/KI^, and 2 males per genotype) derived from *Rap1*^KI/KI^ relative to WT as determine using multiplex cytokine analysis. P values were determined by a two-way ANOVA. **(B)** Individual cytokines including IL-12p40, IL-17, and IL-18 were elevated in serum from *Rap1*^KI/KI^. Graphs show the fold expression of cytokines derived from *Rap1*^KI/KI^ relative to WT. P values were determined by student’s unpaired t-tests. **(C)** The fold expression of cytokines in *Rap1*^KI/KI^ serum relative to WT serum from young and old mice (n = 3 young/middle-aged females per genotype, 2 old WT females, 7 old KI/KI females, 2 young/middle-aged males per genotype, and 3 old male mice per genotype). **(D-E)** Individual cytokines/chemokines CCL3 in the spleen (*n* = 6 WT females, 6 *Rap1*^KI/KI^ females, 3 WT males, and 3 *Rap1*^KI/KI^ males) and RANTES in the lung (*n* = 6 WT females, 6 *Rap1*^KI/KI^ females, 3 WT males, and 3 *Rap1*^KI/KI^ males) were elevated in *Rap1*^KI/KI^ compared to WT tissues. P values were determined by student’s unpaired *t*-tests. Data are mean ± SEM in all graphs. WT was set to 1 in A-E with KI/KI values displayed as relative to the mean WT value for each graph.
(TIF)

S8 Fig *Rap1* knockin mice show signs of metabolic dysfunction. **(A-D)** Mice fed with a standard chow diet. Body weights were assessed weekly over time for females and males. Food intake was quantified weekly in females and males. n = 8 WT females, n = 6 *Rap1*^*KI/WT*^ females, n = 5 *Rap1*^KI/KI^ females. n = 5 males for each genotype. **(E-H)** Mice fed with a high fat high sugar (HFHS) diet. Body weights were assessed weekly starting at ~2 months of age for females and males. Fasting blood glucose was measured in females and males at age 2–4 months. n = 6 WT females, n = 7 *Rap1*^*KI/WT*^ females, n = 4 *Rap1*^KI/KI^ females. n = 5 WT males, n = 3 *Rap1*^*KI/WT*^ males, n = 5 *Rap1*^KI/KI^ males. Food intake p-values were assessed by one-way ANOVAs. Two-way ANOVAs with post-hoc turkey tests were performed for all other experiments. All data are mean ± SEM.
(TIF)

S9 Fig *Rap1* knockin mice show no significant differences in behavior associated with learning/memory. **(A)** Light-dark box test, *n* = 13 WT (8 females and 5 males) and *n = 11 Rap1*^KI/KI^ (5 females and 6 males) mice. p = 0.170 (genotype/sex interaction). **(B)** Y-maze test shows percentage of alternations to new arms of the maze in *Rap1*^KI/KI^ relative to WT mice, *n = 13* WT (8 females and 5 males) and *n = 12 Rap1*^KI/KI^ (5 females and 6 males) mice. p = 0.917. **(C)** Water T-maze test shows percentage of incorrect arm entries in *Rap1*^KI/KI^ relative to WT littermates. *n* = 8 WT (3 females and 5 males) and *n = 13 Rap1*^KI/KI^ (9 females and 4 males) mice. p = 0.190. Behavior results were analyzed in R software with 2-way ANOVA using genotype and sex as factors, with covariates of wave and body weight included where appropriate. All data are mean ± SEM.
(TIF)

S10 Fig Expression of inflammasome pathway components is increased in *Rap1* knockin brain tissues. Representative images of western blots (left panel) and bar graph quantifications (right panel) show expression of the inflammasome pathway components: pro-CASPASE-1 (pro-CASP-1, *n* = 6 WT females, 6 KI/KI females, 7 WT males, 6 KI/KI males), cleaved CASPASE-1 (CASP-1p20, *n* = 6 WT females, 6 KI/KI females, 7 WT males, 6 KI/KI males), NEK7 (*n* = 3 WT females, 6 KI/KI females, 7 WT males, 6 KI/KI males), pro-IL-18 (*n* = 3 WT females, 6 KI/KI females, 7 WT males, 6 KI/KI males), IL-18 (active, *n* = 3 WT females, 6 KI/KI females, 7 WT males, 7 KI/KI males), pro-IL-1β (*n* = 4 WT females, 4 KI/KI females, 6 WT males, 5 KI/KI males), and GAPDH (protein loading control) in brain tissue lysates derived from WT and *Rap1*^KI/KI^. Each point represents a biological replicate, and technical replicates were averaged prior to graphing. female mice **(A-B)** and male mice **(C-D)**.
(TIF)

S11 Fig Overlapping deregulated genes in *Rap1* mutant knockin and *Rap1* knockout liver tissues. The Venn diagrams display deregulated genes in *Rap1* null vs. WT livers only (blue), in *Rap1*^KI/KI^ vs. WT livers only (pink), and genes that were deregulated in both *Rap1* null and *Rap1*^KI/KI^ livers (overlap). Overlapping genes are listed. Upregulated genes are red, and downregulated genes are blue. Data from *Rap1* null livers tissues is from the cited sources (above blue diagrams).
(TIF)

S1 Table Gene Transcription is altered in *Rap1*^KI/KI^ tissues. (XLSX)

S2 Table Genetic pathways are altered in *Rap1*^KI/KI^ tissues. (XLSX)

S3 Table Cytokine/Chemokine expression in untreated and LPS-stimulated WT and *Rap1* knockin BMDMs. (XLSX)

S4 Table Cytokine/chemokine expression in WT and *Rap1* knockin serum and tissues. (XLSX)

S5 Table Tumor incidence and gross abnormalities are increased in *Rap1* knockin mice. (XLSX)
