# Supplementary figures and images for: Aberrant expression and localization of the RAP1 shelterin protein contribute to age-related phenotypes
Source: PLoS Genet. 2022 Nov 28;18(11):e1010506. doi: 10.1371/journal.pgen.1010506 (PMC9704629; doi:10.1371/journal.pgen.1010506)

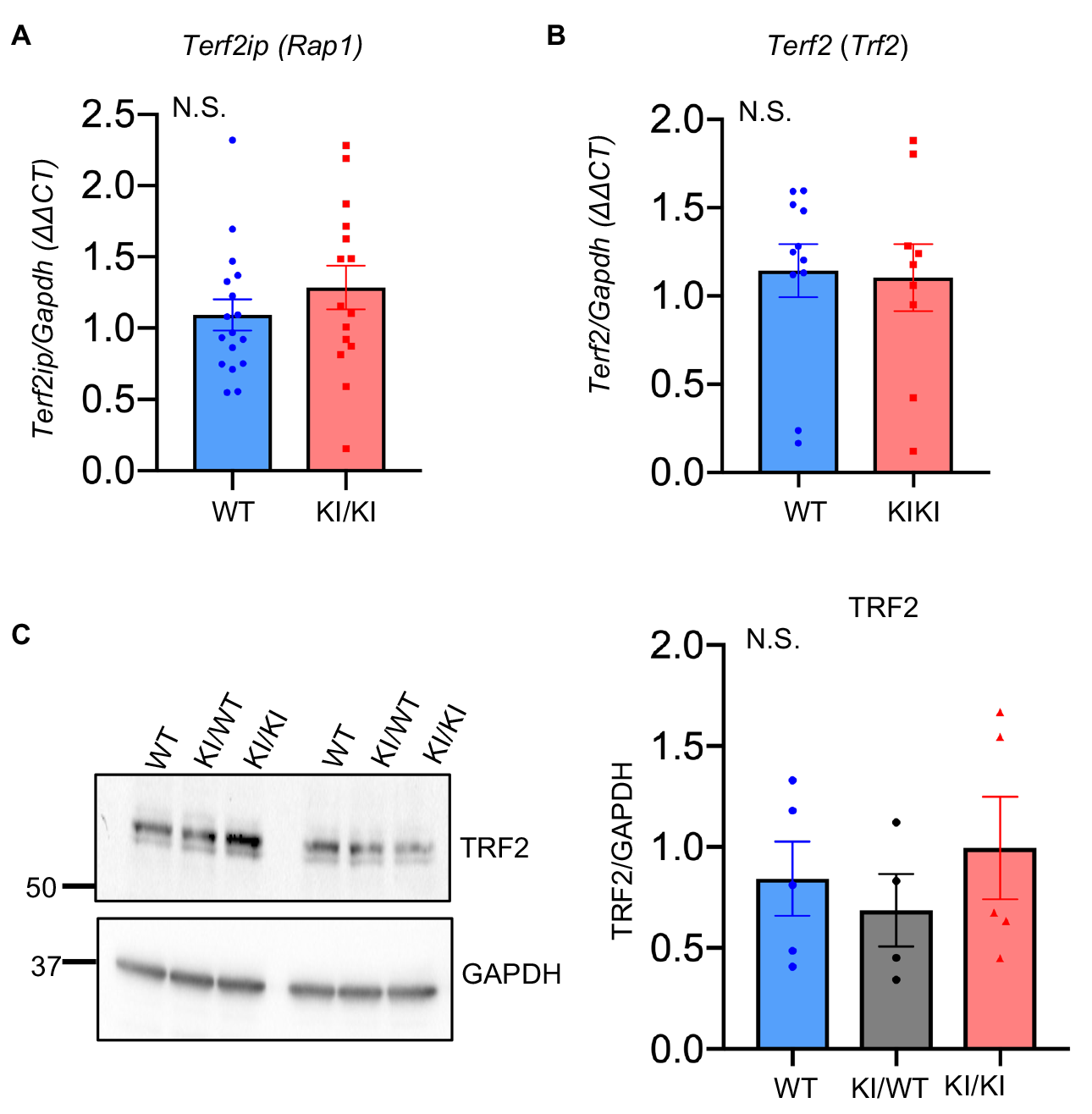

Supplement: S1 Fig — (A-B) Rap1 and Trf2 mRNA expression levels in WT and Rap1KI/KI primary MEFs by RT-qPCR. n = 5 primary MEF cell lines for each genotype with technical duplicates. (C) Western blot on whole-cell lysates from WT, Rap1KI/WT, and Rap1KI/KI primary MEFs. Quantification shows no significant differences in TRF2 protein levels among the genotypes. P values were not significant (N.S.) and determined by a one-way ANOVA with student’s unpaired t-tests (A-B) and Tukey’s post-hoc comparisons (C). Data are mean ± SEM. n = 5 primary MEF cell lines for each genotype. (TIF) [file pgen.1010506.s001.tif]

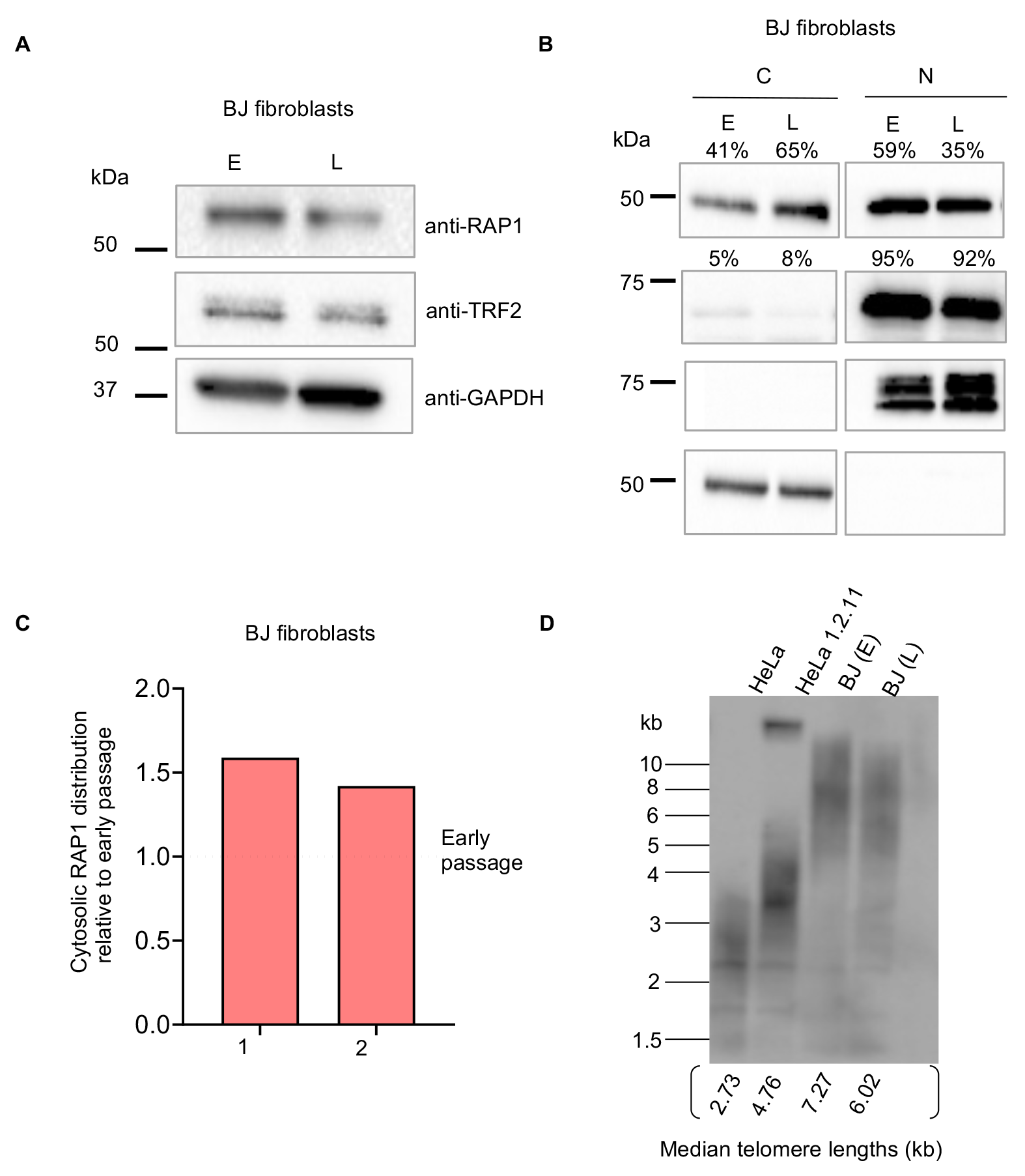

Supplement: S2 Fig — (A) Representative western blot analysis of RAP1 and TRF2 levels using whole-cell lysates derived from primary BJ fibroblasts at early (E) and late (L) passages (non-dividing). (B-C) Representative western blot analysis of RAP1 and TRF2 levels using cytosol (C) and nuclear (N) fractions derived from BJ fibroblasts at early (E) and late (L) passages. Lamin A and tubulin are nuclear and cytosolic proteins, respectively. The percent distribution of RAP1 and TRF2 in each fraction is shown. (D) Telomere length measurement of HeLa, HeLa 1.2.11, early passage (E), and late passage (L) BJ fibroblasts by telomere restriction fragment analysis using a biotin conjugated telomere probe. (TIF) [file pgen.1010506.s002.tif]

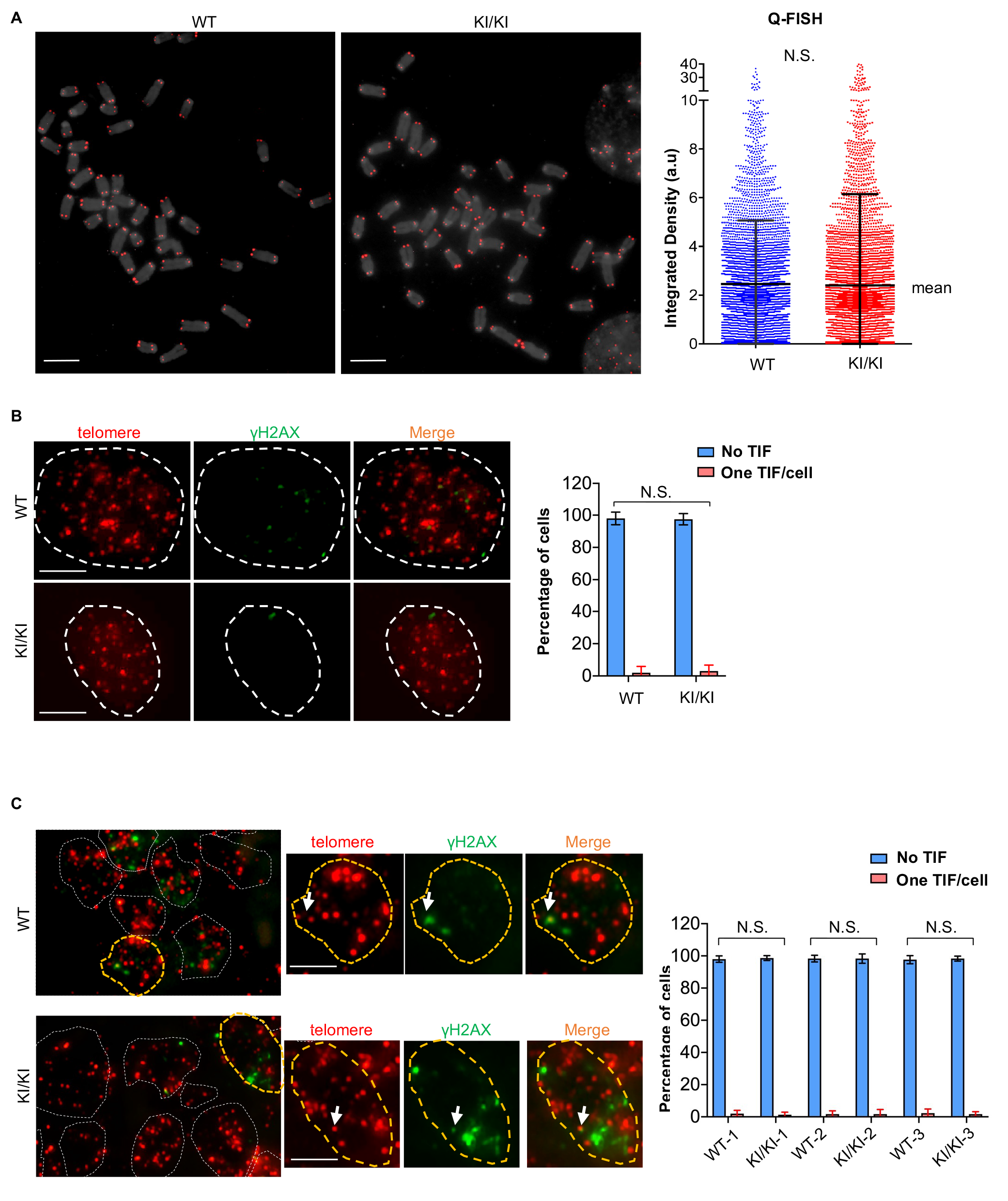

Supplement: S3 Fig — (A) Representative metaphase spreads of Q-FISH analysis on WT and Rap1KI/KI primary MEFs showing DAPI (grey) and telomere signals (red). Quantitative measurement of telomere signal intensities is shown (right panel). Bars represent mean ± SD. Scale bars: 20μm. (B-C) Representative images of TIF analysis by IF-telomere FISH on primary MEFs (B) and mouse spleen tissues (C) using telomere FISH (red) and anti-γH2AX (green). n = 172 WT MEFs, n = 134 Rap1KI/KI MEFs, n = ~300 splenocytes per mouse analyzed. WT and Rap1KI/KI mice are 20, 22, and 24 months. Cells with ≥3 TIFs were not observed. P values between WT and Rap1KI/KI were not significant. Data are mean ± SEM. Scale bars: 5μm. (TIF) [file pgen.1010506.s003.tif]

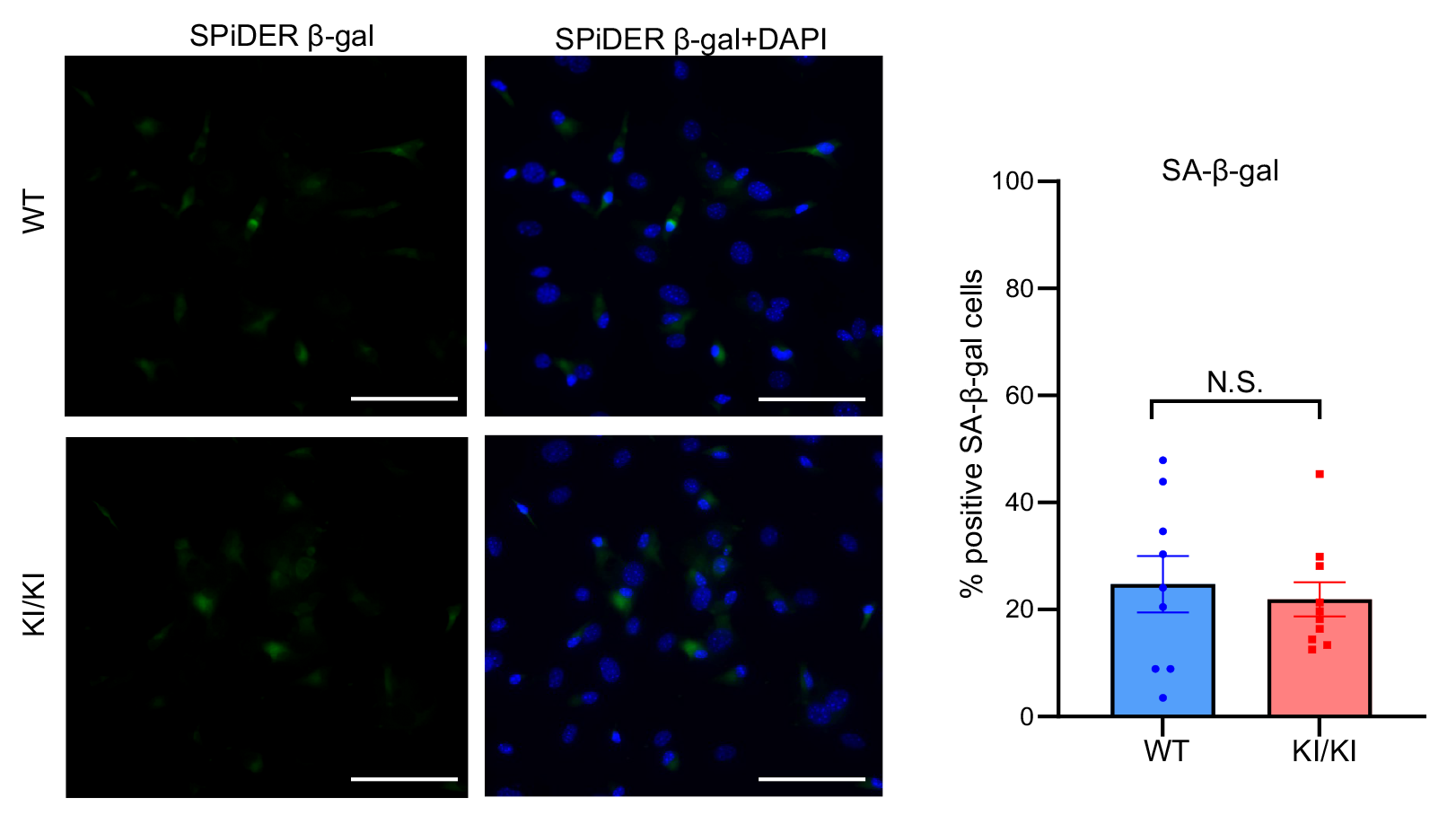

Supplement: S4 Fig — Representative images (left) of primary WT and Rap1KI/KI MEFs stained with SPiDER-β-gal (green) and DAPI (blue). A minimum of 8 images containing ~300 cells were captured. The right panel shows plotted data points for individual images, and each data point was derived from an image. MEFs were at passage 5. Scale bars: 50μm. Data are mean ± SEM. (TIF) [file pgen.1010506.s004.tif]

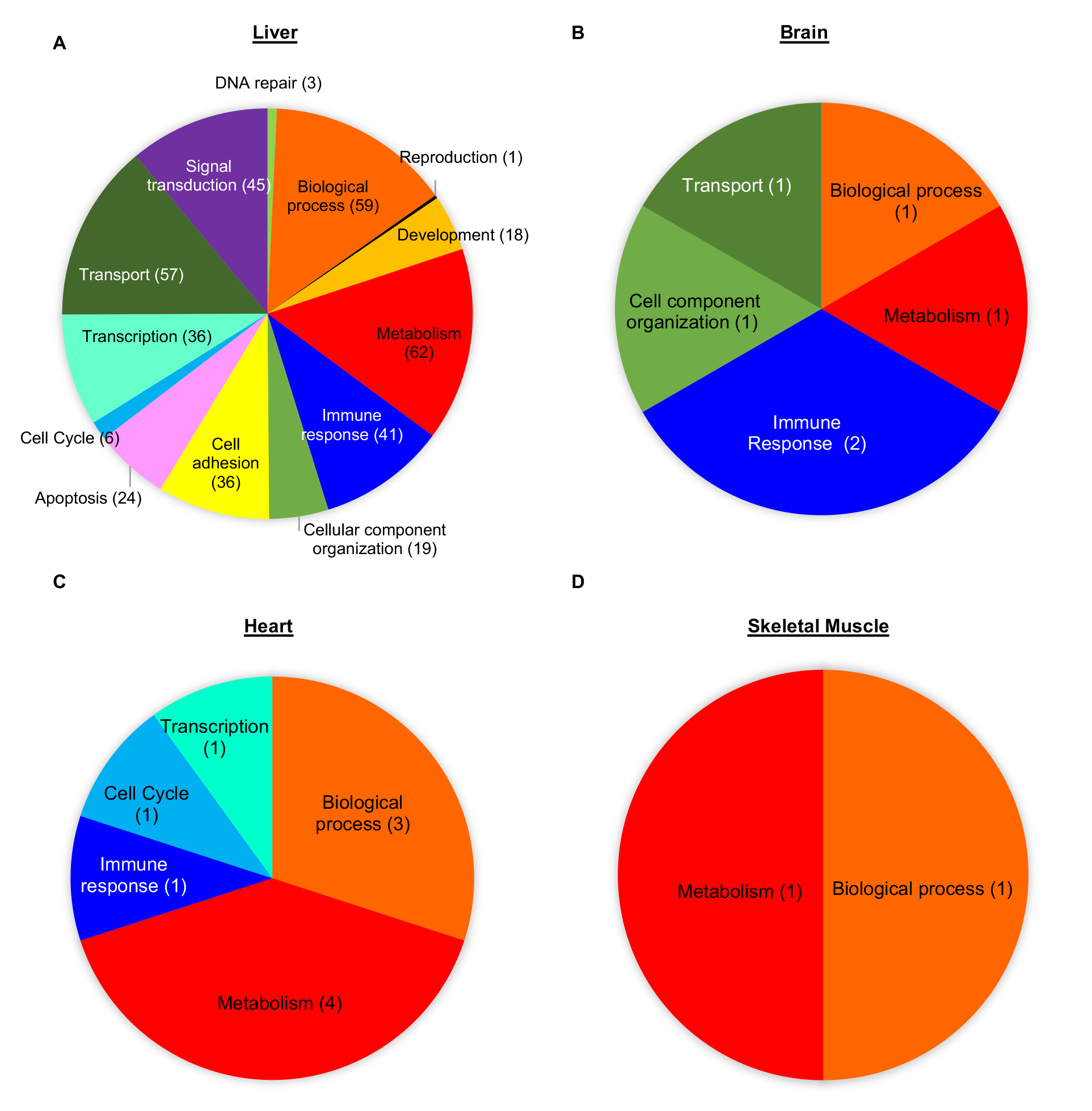

Supplement: S5 Fig — Gene ontology classifications for the significantly altered transcripts as determined by microarray analysis in the liver (A), brains (B), hearts (C), and skeletal muscles (D) of Rap1KI/KI compared to WT mice. n = 3 mice per genotype. (TIF) [file pgen.1010506.s005.tif]

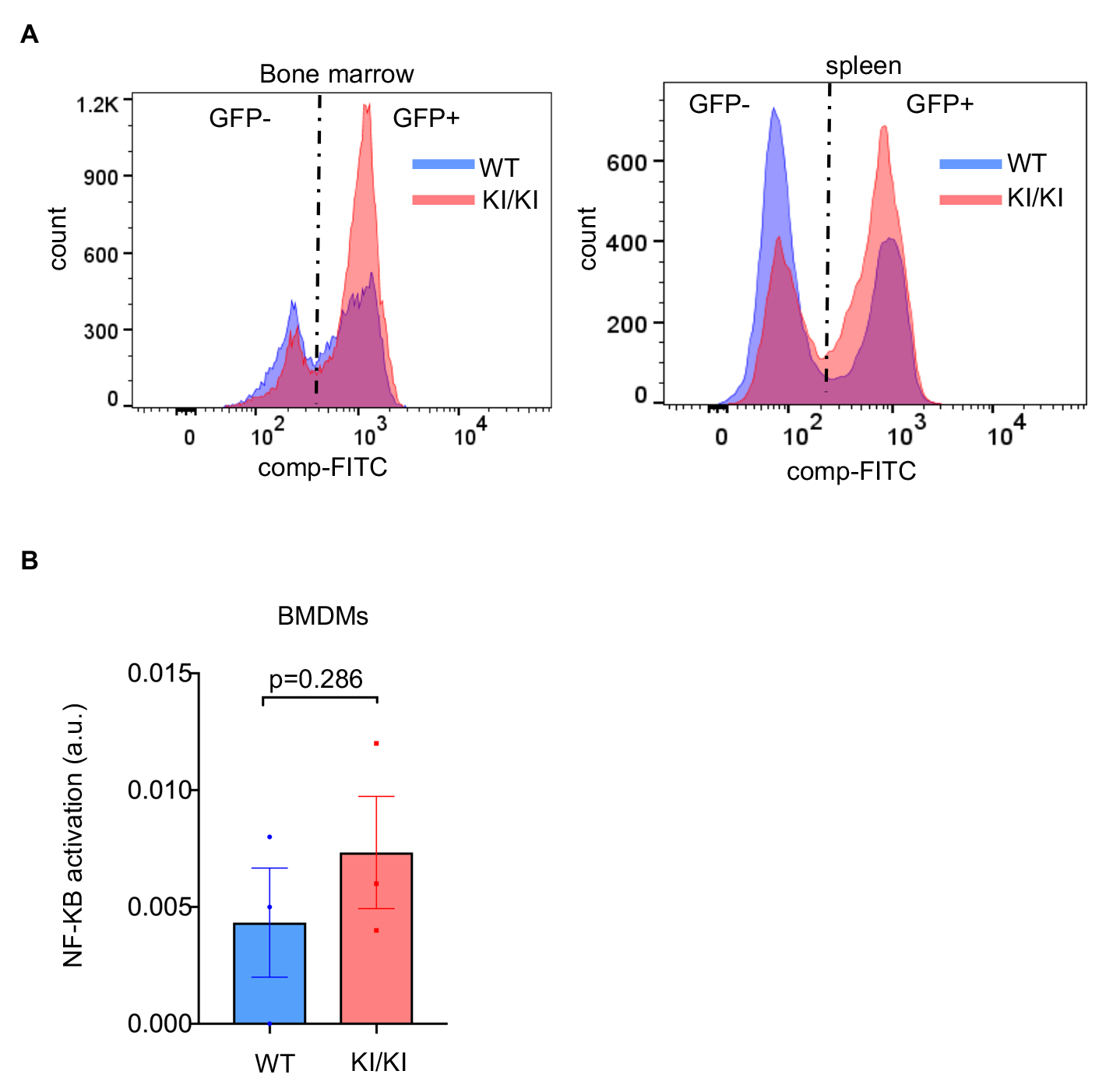

Supplement: S6 Fig — (A) Histograms represent the population of GFP-positive cells (GFP+) in bone marrow (left) and splenocytes (right) derived from WT (blue histograms) or Rap1KI/KI (red histograms) mice in RelA-EGFP reporter background by flow cytometry analysis. Gating for GFP+ cells is indicated by the dotted line. n = 5 WT and n = 3 Rap1KI/KI mice. (B) Bar graph shows NF-κB activation as determined by the level of p65 expressed in nuclear extracts derived from WT and Rap1KI/KI mouse BMDMs using the NF-κB p65 transcription factor assay kit. P values were determined using student’s unpaired t-tests. All data are mean ± SEM. (TIF) [file pgen.1010506.s006.tif]

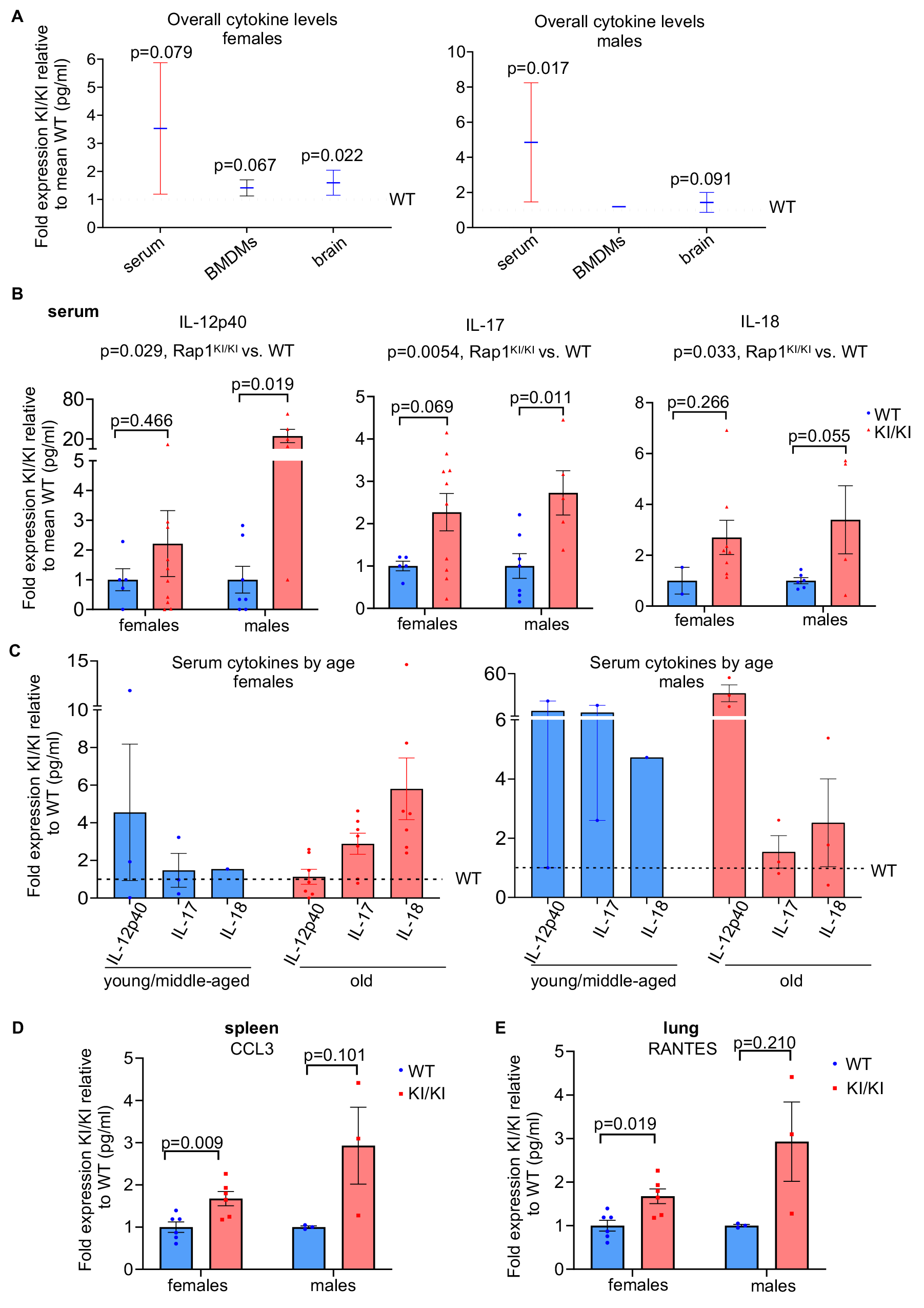

Supplement: S7 Fig — (A) The overall levels of cytokines and chemokines were significantly elevated in serum (n = 5 WT female, 10 Rap1KI/KI females, 7 WT males, and 5 Rap1KI/KI male mice), BMDMs (n = 2 WT females, 2 Rap1KI/KI females, and 1 male per genotype), and brain tissues (n = 5 WT female, 9 female Rap1KI/KI, and 2 males per genotype) derived from Rap1KI/KI relative to WT as determine using multiplex cytokine analysis. P values were determined by a two-way ANOVA. (B) Individual cytokines including IL-12p40, IL-17, and IL-18 were elevated in serum from Rap1KI/KI. Graphs show the fold expression of cytokines derived from Rap1KI/KI relative to WT. P values were determined by student’s unpaired t-tests. (C) The fold expression of cytokines in Rap1KI/KI serum relative to WT serum from young and old mice (n = 3 young/middle-aged females per genotype, 2 old WT females, 7 old KI/KI females, 2 young/middle-aged males per genotype, and 3 old male mice per genotype). (D-E) Individual cytokines/chemokines CCL3 in the spleen (n = 6 WT females, 6 Rap1KI/KI females, 3 WT males, and 3 Rap1KI/KI males) and RANTES in the lung (n = 6 WT females, 6 Rap1KI/KI females, 3 WT males, and 3 Rap1KI/KI males) were elevated in Rap1KI/KI compared to WT tissues. P values were determined by student’s unpaired t-tests. Data are mean ± SEM in all graphs. WT was set to 1 in A-E with KI/KI values displayed as relative to the mean WT value for each graph. (TIF) [file pgen.1010506.s007.tif]

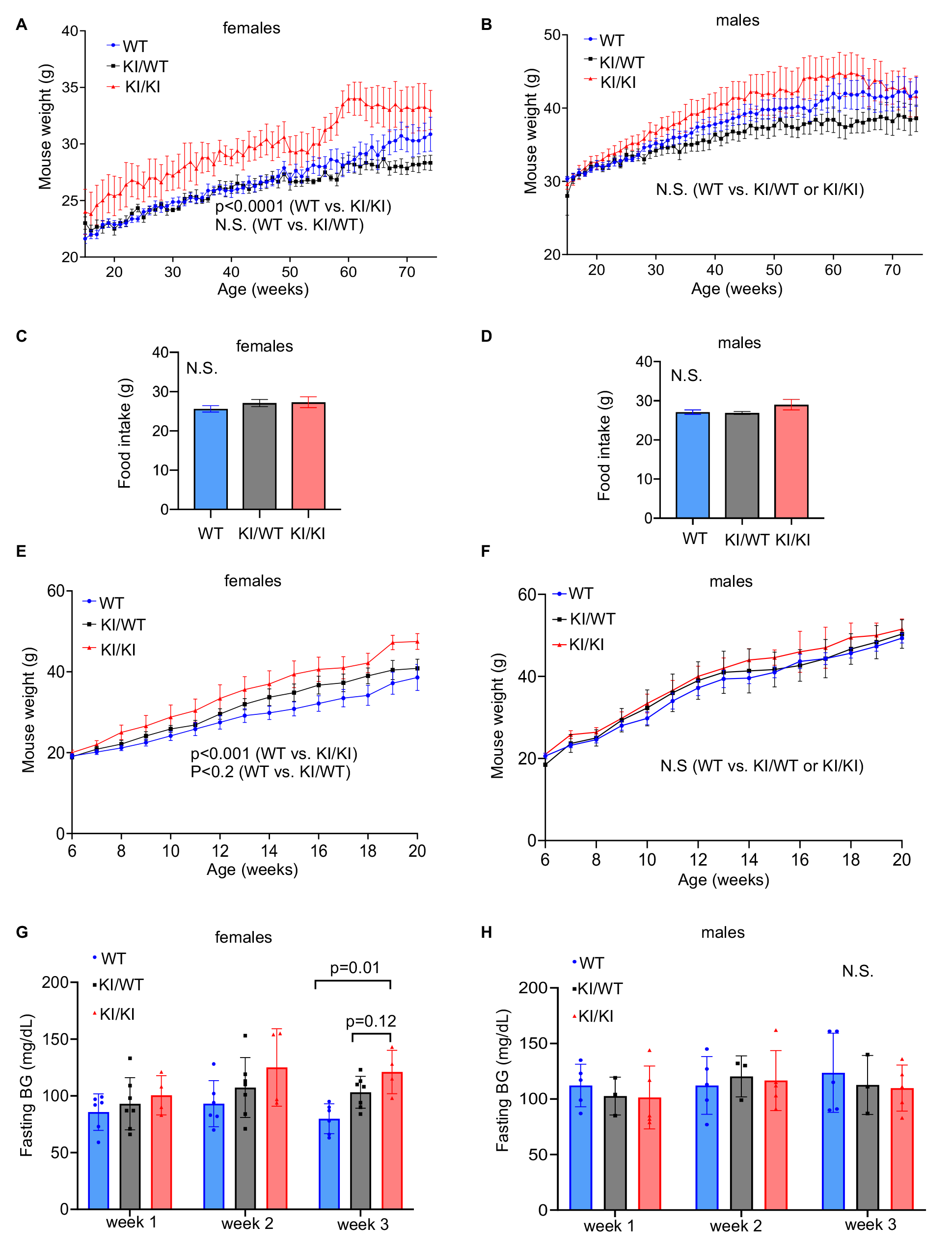

Supplement: S8 Fig — (A-D) Mice fed with a standard chow diet. Body weights were assessed weekly over time for females and males. Food intake was quantified weekly in females and males. n = 8 WT females, n = 6 Rap1KI/WT females, n = 5 Rap1KI/KI females. n = 5 males for each genotype. (E-H) Mice fed with a high fat high sugar (HFHS) diet. Body weights were assessed weekly starting at ~2 months of age for females and males. Fasting blood glucose was measured in females and males at age 2–4 months. n = 6 WT females, n = 7 Rap1KI/WT females, n = 4 Rap1KI/KI females. n = 5 WT males, n = 3 Rap1KI/WT males, n = 5 Rap1KI/KI males. Food intake p-values were assessed by one-way ANOVAs. Two-way ANOVAs with post-hoc turkey tests were performed for all other experiments. All data are mean ± SEM. (TIF) [file pgen.1010506.s008.tif]

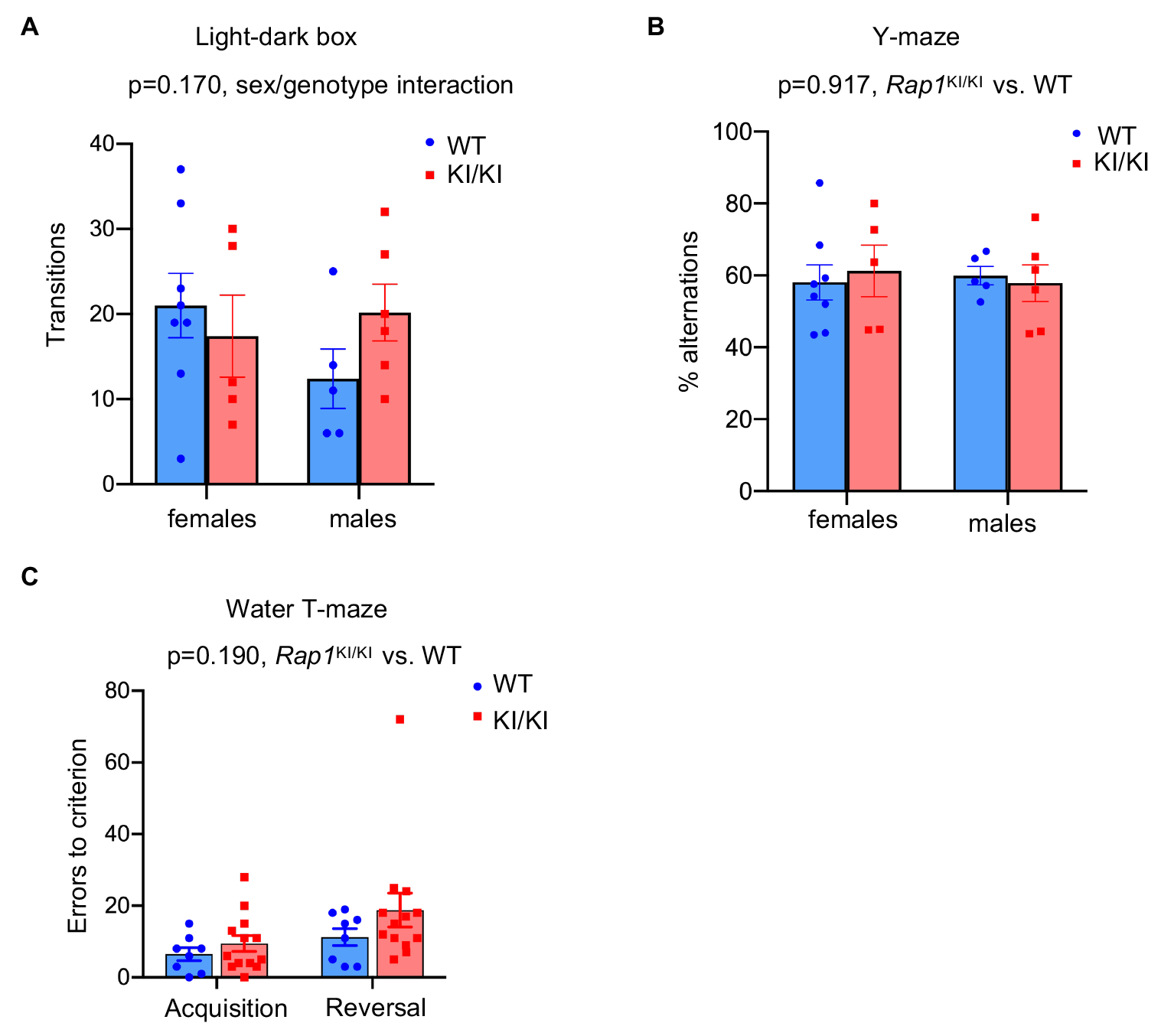

Supplement: S9 Fig — (A) Light-dark box test, n = 13 WT (8 females and 5 males) and n = 11 Rap1KI/KI (5 females and 6 males) mice. p = 0.170 (genotype/sex interaction). (B) Y-maze test shows percentage of alternations to new arms of the maze in Rap1KI/KI relative to WT mice, n = 13 WT (8 females and 5 males) and n = 12 Rap1KI/KI (5 females and 6 males) mice. p = 0.917. (C) Water T-maze test shows percentage of incorrect arm entries in Rap1KI/KI relative to WT littermates. n = 8 WT (3 females and 5 males) and n = 13 Rap1KI/KI (9 females and 4 males) mice. p = 0.190. Behavior results were analyzed in R software with 2-way ANOVA using genotype and sex as factors, with covariates of wave and body weight included where appropriate. All data are mean ± SEM. (TIF) [file pgen.1010506.s009.tif]

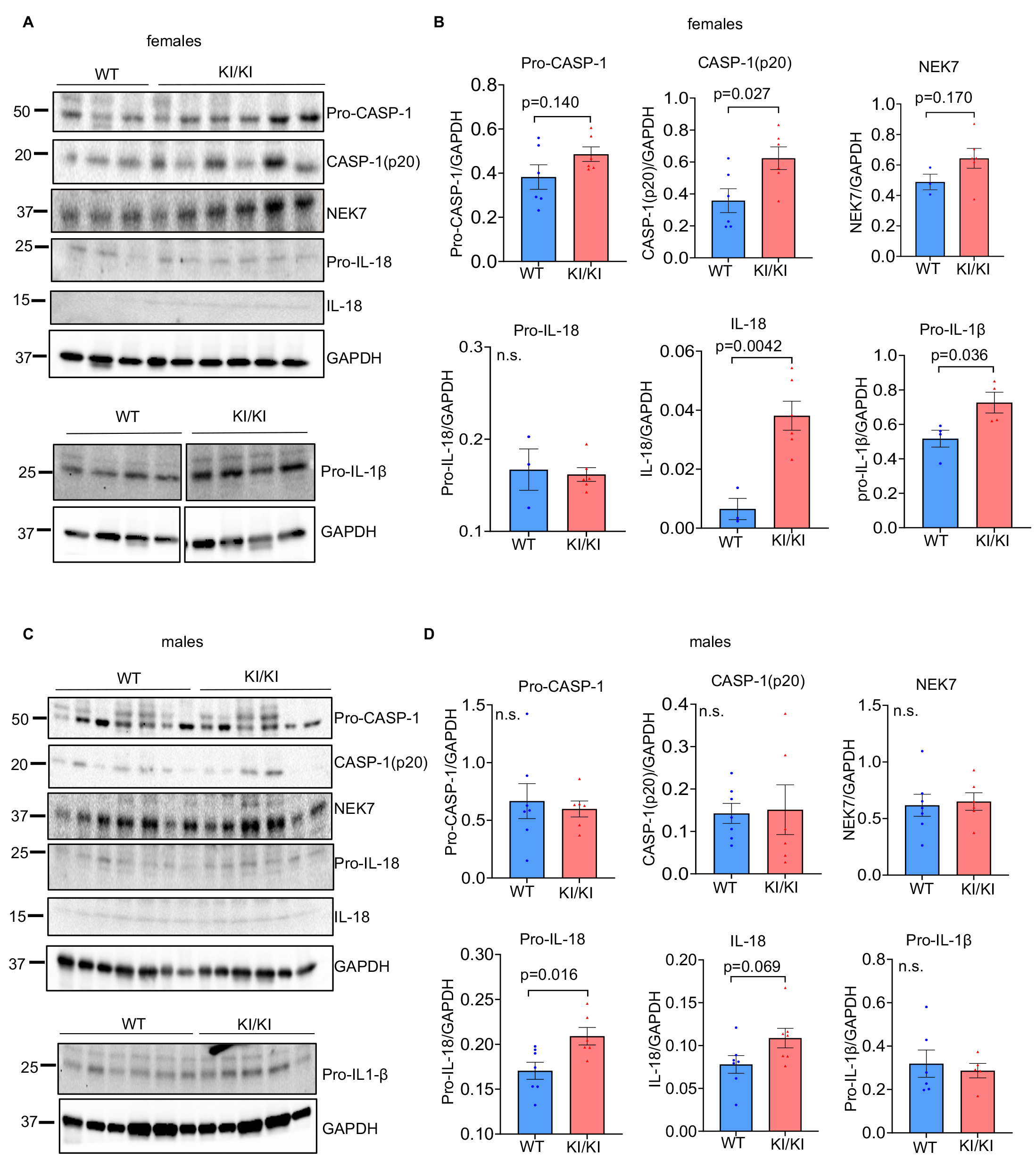

Supplement: S10 Fig — Representative images of western blots (left panel) and bar graph quantifications (right panel) show expression of the inflammasome pathway components: pro-CASPASE-1 (pro-CASP-1, n = 6 WT females, 6 KI/KI females, 7 WT males, 6 KI/KI males), cleaved CASPASE-1 (CASP-1p20, n = 6 WT females, 6 KI/KI females, 7 WT males, 6 KI/KI males), NEK7 (n = 3 WT females, 6 KI/KI females, 7 WT males, 6 KI/KI males), pro-IL-18 (n = 3 WT females, 6 KI/KI females, 7 WT males, 6 KI/KI males), IL-18 (active, n = 3 WT females, 6 KI/KI females, 7 WT males, 7 KI/KI males), pro-IL-1β (n = 4 WT females, 4 KI/KI females, 6 WT males, 5 KI/KI males), and GAPDH (protein loading control) in brain tissue lysates derived from WT and Rap1KI/KI. Each point represents a biological replicate, and technical replicates were averaged prior to graphing. female mice (A-B) and male mice (C-D). (TIF) [file pgen.1010506.s010.tif]

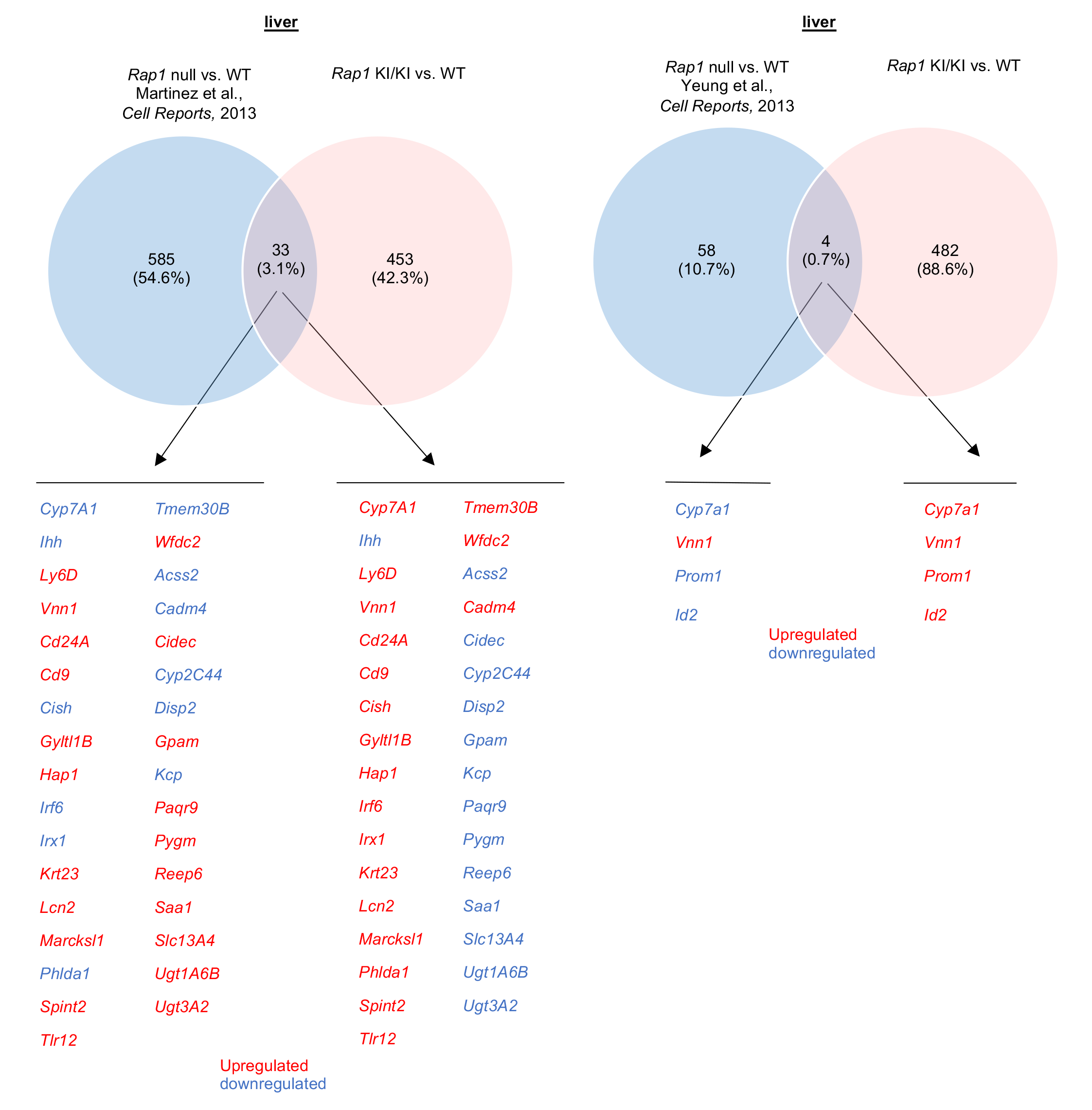

Supplement: S11 Fig — The Venn diagrams display deregulated genes in Rap1 null vs. WT livers only (blue), in Rap1KI/KI vs. WT livers only (pink), and genes that were deregulated in both Rap1 null and Rap1KI/KI livers (overlap). Overlapping genes are listed. Upregulated genes are red, and downregulated genes are blue. Data from Rap1 null livers tissues is from the cited sources (above blue diagrams). (TIF) [file pgen.1010506.s011.tif]
